# Current Challenges in Hemostasis and Advances in Particle‐Assisted Styptic Devices

**DOI:** 10.1002/adhm.202502600

**Published:** 2025-10-05

**Authors:** Daniele Baiocco, Zhibing Zhang, Neil M. Eisenstein, Nigel Tai, Liam M. Grover

**Affiliations:** ^1^ School of Chemical Engineering University of Birmingham Birmingham B15 2TT UK; ^2^ Healthcare Technology Institute Institute of Translational Medicine Queen Elizabeth Hospital University of Birmingham Birmingham B15 2TH UK; ^3^ Research and Clinical Innovation Royal Centre for Defence Medicine ICT Centre Vincent Drive Birmingham B15 2SQ UK; ^4^ Academic Department of Military Surgery and Trauma Royal Centre for Defence Medicine Birmingham B15 2TH UK

**Keywords:** biomimetic approaches, blood clotting control, hemostatic devices, microcapsules, microparticles

## Abstract

Rapid and effective hemorrhage control is critical in trauma and surgical interventions, where uncontrolled bleeding remains a leading cause of preventable death. In response to this urgent clinical demand, the development of novel hemostatic materials is the focus of increasing research interest, in both academia and industry. Styptic dressings are gradually evolving to address this need. However, significant challenges, such as delayed activation, suboptimal performance in severe conditions, and biocompatibility issues, persist. Here current limitations in hemostatic dressing technologies are explored, and recent innovations including biomimetic approaches in this field are highlighted. Special emphasis is placed on microparticle‐integrated and nanoengineered systems integrated with drug delivery technologies. By addressing these challenges, it is aimed to inspire new pathways for the development of next‐generation multifunctional dressings with enhanced efficacy and accessibility.

## Graphical abstract

Here persistent limitations in hemostatic technologies are highlighted and cutting‐edge biomimetic, microparticle‐assisted, and nanoengineered systems with integrated drug delivery are showcased. Moreover, the article identifies fresh directions toward the next‐generation of multifunctional hemostatic devices with superior efficacy and accessibility, critically assessing novel strategies to overcome activation delays, performance gaps, and biocompatibility issues.

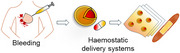

## 1 Introduction

Uncontrolled bleeding, often resulting from motor vehicle accidents, interpersonal violence, large‐scale conflict, or natural catastrophe, is one of the primary causes of mortality in trauma patients, particularly in prehospital settings.^[^ ^1^ ^]^ Rapid and effective hemorrhage control not only saves lives but also improves patients’ health by minimizing subsequent complications, such as hypovolemic shock, organ failure, and life‐changing disabilities.^[^ ^2^ ^]^ In response to these needs, over the years, hemostatic dressings have become indispensable in both military and civilian healthcare settings due to their ease of use and handling, promoting blood clotting, and enhancing wound management.^[^ ^3^ ^]^

However, conventional hemostatic dressings face significant drawbacks in complex clinical scenarios, including delayed activation, reduced performance in coagulopathic patients, and risks of adverse reactions, such as inflammation and/or bacterial infection.^[^ ^4^, ^5^ ^]^

To circumvent these limitations, advances in materials science and nanotechnology are driving the fabrication of smart, on‐demand, stimuli‐responsive hemostatic wound devices (HWDs), capable of activating in response to physiological triggers such as temperature, pH, or enzymatic activity of body fluids.^[^ ^6^ ^]^ Within the field, cutting‐edge innovations aim to optimize the performance of HWDs and their broad‐spectrum functionalities (e.g., antimicrobial and tissue regeneration properties), thereby expediting the overall wound‐healing process.^[^ ^3^ ^]^ To this end, new organic, inorganic, and bioinspired materials—or a blend thereof—are emerging as products designed to achieve more rapid hemostasis.^[^ ^7^ ^]^ Among these, micro/nanocarriers are being actively investigated for the delivery of biological and nonbiological molecules for hemostasis and rapid cell repair.^[^ ^8^ ^]^

Microparticle‐integrated and nanoengineered systems represent a transformative leap in hemostatic technology by enabling rapid, site‐specific delivery of pro‐thrombogenic bioactive agents, promoting faster coagulation under complex conditions.^[^ ^9^ ^]^ Their tuneable surface chemistry, high surface‐area‐to‐volume ratio, and stimuli responsiveness offer clear advantages over conventional dressings.^[^ ^10^ ^]^ However, challenges such as manufacturing scalability, regulatory hurdles, and long‐term safety remain significant.

This review critically examines the contemporary landscape of HWDs, highlighting critical challenges and novel strategies for transformative innovation. We explore how emerging materials and mechanisms, informed by advancements in nanotechnology and bioengineering, are transforming this dynamic field. Furthermore, we identify key unmet clinical needs and propose a forward‐looking roadmap to guide researchers and practitioners in advancing hemostatic technologies to fulfill evolving demands.

## 2 The Multifaceted Intersection of Hemostasis: Conflicts, Economics, and Medicine

### 2.1 Modern Warfare

In modern warfare and humanitarian crises, hemorrhage remains the leading cause of preventable death, exacerbated by intentional/nonintentional attacks on healthcare infrastructure.^[^ ^11^ ^]^ The conflict in Ukraine has demonstrated the devastating consequences of targeted and indiscriminate offensives on medical facilities, with over 1500 raids on healthcare, some 800 hospitals and medical resources damaged or destroyed and more than 250 health workers killed.^[^ ^12^ ^]^ Similar scenarios have been reported in the North Gaza governorate.^[^ ^13^ ^]^ Such attacks upon medical facilities reduce the timely availability of surgical/nonsurgical hemorrhage control, catastrophically delaying patient evacuation and accessibility to treatment against life‐threatening hemorrhage.^[^ ^14^ ^]^ Lessons from past military operations in Afghanistan and Iraq highlight the importance of educating the public, disseminating bleeding control training, even for laypersons without medical backgrounds, using principles espoused within Tactical Combat Casualty Care (TCCC) guidelines. TCCC emphasizes the role of tourniquets in life‐threatening extremity hemorrhage.^[^ ^11^, ^15^ ^]^ However, while bleeding control kits have been developed for use in public spaces, such measures have yet to be universally adopted.

### 2.2 Geoeconomic Views on Hemostatic Agents

From a geoeconomic perspective, the hemostatic agent market is experiencing rapid expansion, driven by military demand and increased trauma cases. The global market for topical hemostatic dressings alone was estimated just under USD 3 billion in 2023, with a compound annual growth rate (CAGR) of 6–7% between 2024 and 2030.^[^ ^16^ ^]^

Military forces, emergency response organizations, and civilian healthcare systems are increasingly investing in next‐generation biomaterials for the management of wounds, including particle‐based hemostatics and self‐expanding foams, which could further improve prehospital trauma care.^[^ ^17^, ^18^ ^]^ However, economic disparities between nations mean that access to these potentially life‐saving tools is uneven, disproportionately affecting low‐resource settings where trauma mortality rates are highest.^[^ ^19^ ^]^ Global instability, rise in interpersonal violence, increasing use of motorized transportation among low‐ and middle‐income countries, and actual or impending societal conflict is likely to accelerate the requirement for novel hemorrhage control technologies.

### 2.3 Ecosystem of Hemostasis

Hemostasis can be approached through physical, surgical, and pharmacological means, each with advantages and limitations depending on the specific case.^[^ ^20^ ^]^ Nonsurgical physical methods, such as tourniquets, are effective for rapid stabilization but come with risks of distal ischemia, vascular damage, nerve palsy, and reperfusion injury if left in place for too long.^[^ ^21^ ^]^ Other emerging approaches, such as intracavitary expanding foam, are under investigation with promising animal data but requires more extensive clinical evidence.^[^ ^22^ ^]^

Additional methods like resuscitative endovascular balloon occlusion of the aorta (REBOA) provide effective temporization of exsanguination from noncompressible torso hemorrhage (NCTH) in the abdomen or pelvis.^[^ ^23^ ^]^ However, REBOA creates considerable lower‐body ischemia if balloon inflation times are prolonged, with risk of significant complications and may be worse than standard care in some settings.^[^ ^24^ ^]^

Surgical interventions, including vascular clamping, shunting, and repair, remain the definitive solution for major hemorrhage.^[^ ^25^ ^]^ However, these require equipped surgical facilities and specialized personnel, both of which are often unavailable in austere settings.

**Figure 1:**
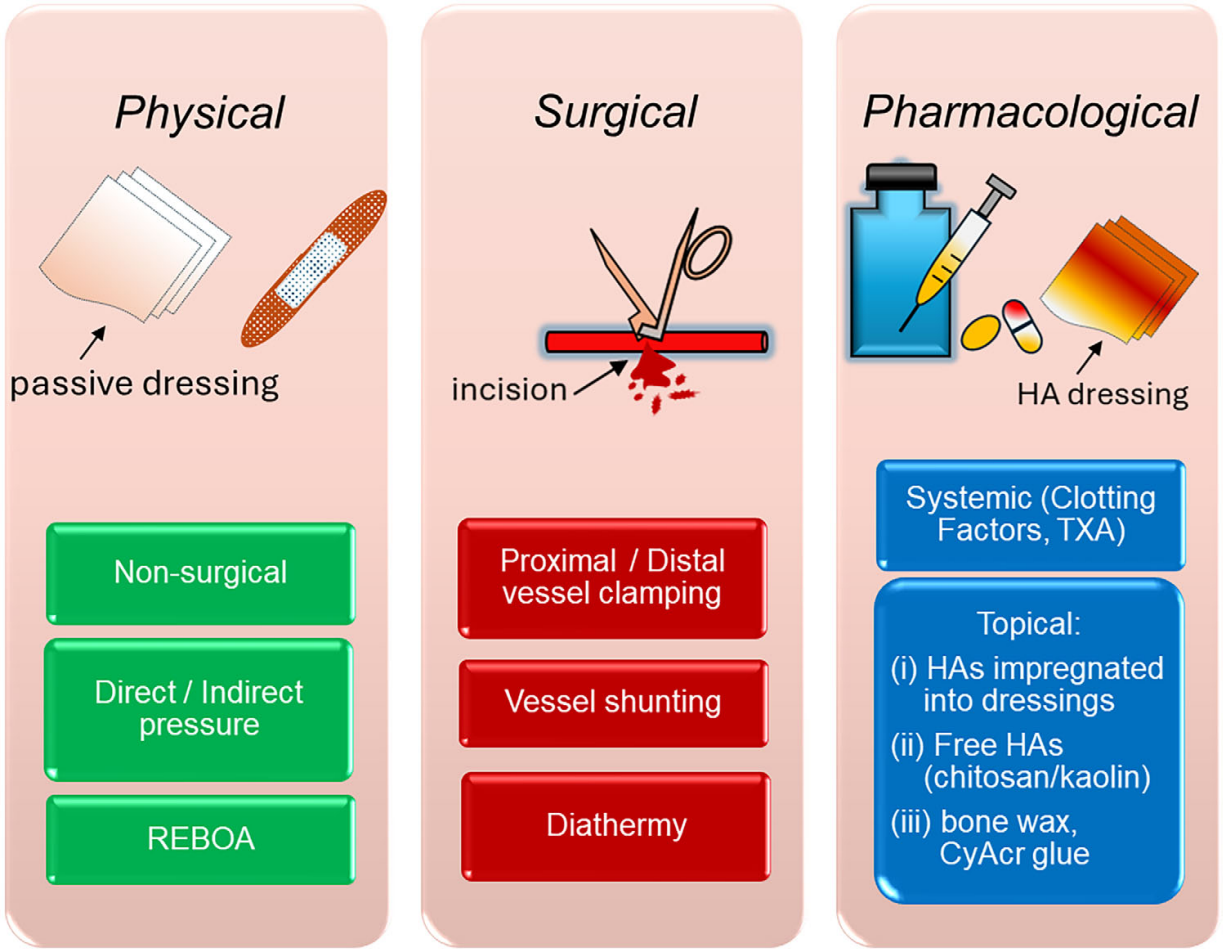
Roadmap of the ecosystem of hemostasis. The figure categorizes hemostasis strategies into three primary approaches: physical, surgical, and pharmacological. i) Physical methods include nonsurgical techniques such as direct and indirect pressure, as well as advanced interventions like Resuscitative Endovascular Balloon Occlusion of the Aorta (REBOA). ii) Surgical techniques involve mechanical interventions such as proximal/distal vessel clamping, vessel shunting, and diathermy for cauterization. iii) Pharmacological approaches comprise systemic agents (e.g., broad spectrum clotting factors and tranexamic acid (TXA)) and topical hemostatic agents (HA) applied via dressings, granular forms (e.g., chitosan/kaolin), or specialized adhesives like bone wax and cyanoacrylate (CyAcr) glue. Generated using Microsoft Office 365.

The pharmacological approach includes systemic interventions like tranexamic acid (TXA; monocarboxylic acid) which acts as a clot stabilizer through its antifibrinolytic action. The administration of TXA has proven effective at reducing fatalities from trauma‐related hemorrhage.^[^ ^26^ ^]^ Pharmacodynamically, TXA helps reduce blood clot breakdown by inhibiting plasminogen activation and its subsequent conversion into plasmin.^[^ ^27^ ^]^ Other topical, pharmacological hemostatic agents, whether incorporated into dressings (e.g., Celox gauze and QuickClot gauze) or applied as free granules (e.g., chitosan and kaolin‐based products), are emerging as a crucial tool in battlefield medicine and prehospital care.^[^ ^28^ ^]^ Although these agents are relatively inexpensive and can provide control of external hemorrhage, they have potential drawbacks. QuikClot may cause thermal injury, while Celox carries a risk of granule migration into the bloodstream.^[^ ^29^ ^]^ Additionally, frequent mechanical change of the dressing can impair wound healing, increasing the risk of infections (see Sections 4.1–4.3).^[^ ^30^ ^]^ A roadmap illustrating the “ecosystem of hemostasis” is presented in **Figure** **1**.

## 3 Traditional Wound Management and Its Limitations

Understanding the wound‐healing process is critical for evaluating the efficacy of hemostatic agents. While traditional dressings are broadly used, they often lack the bioactivity needed to support key healing stages, including inflammation resolution and tissue regeneration. This section examines the physiology of wound healing and its implications.

### 3.1 Anatomy of Skin and Physiology of Wound Healing

**Figure 2:**
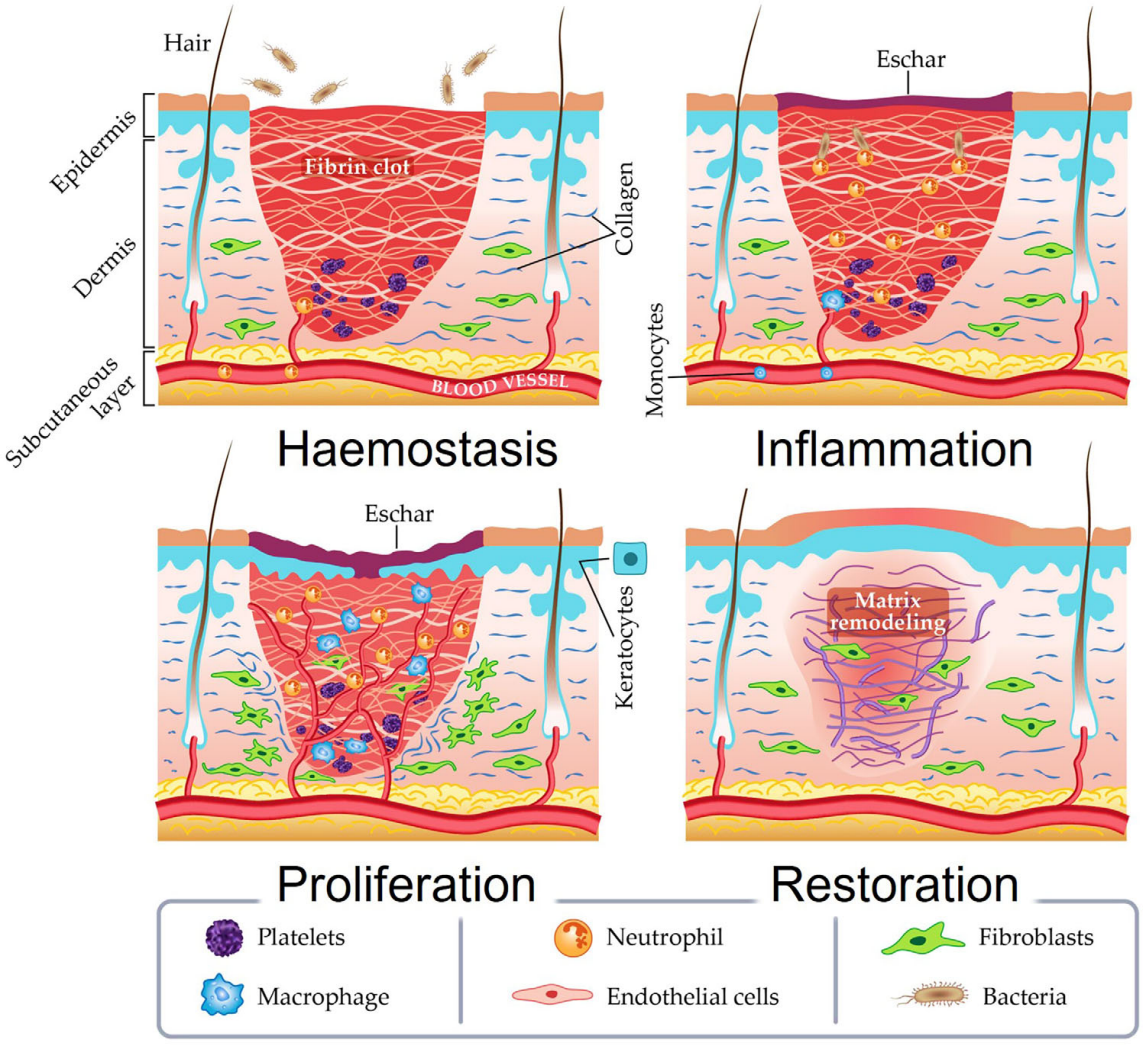
Three‐layer composition of human skin, four‐stages of wound‐healing mechanism (hemostasis, inflammation, proliferation, and remodeling), and key major cellular components, including the key mediators (platelets, neutrophils, fibroblasts, etc.). Reproduced with permission under the terms of the Creative Commons CC BY license 4.0.^[^ ^32^ ^]^ Copyright 2018, Negut et al., published by MDPI.

The skin, the largest organ of the human body, serves as a primary barrier against harmful microorganisms, physical injury, and dehydration.^[^ ^31^ ^]^ Structurally, it is composed of three layers (**Figure** **2**): i) the outer epidermis, which prevents water loss and shields against environmental threats; ii) the dermis, made of connective tissue, which supports the epidermis and plays a pivotal role in wound healing; and iii) the hypodermis (subcutaneous layer or *fascia*), composed of adipose tissue, which provides thermal insulation and absorbs mechanical shocks against bones and muscles.^[^ ^32^, ^33^ ^]^

When injured, the skin initiates a complicated physiological wound‐healing process (Figure 2) comprising four distinct, consecutive stages: i) hemostasis (acute phase with active bleeding points), (ii) inflammation (postacute phase following injury), iii) proliferation (intermediate phase involving granulation tissue formation, angiogenesis, and epithelialisation), and iv) remodeling/restoration (long‐term phase marked by scar tissue formation).^[^ ^32^, ^33^, ^34^ ^]^

This process is highly sensitive and influenced by factors such as wound type, depth, and environmental conditions. While minor injuries can often be managed with home remedies, severe wounds may result in profuse blood loss.^[^ ^35^ ^]^ Traditional wound care approaches often struggle to effectively manage external hemorrhage and are sometimes inadequate for fully restoring skin functionality due to challenges such as dehydration, bacterial proliferation, and suboptimal wound environments.^[^ ^6^, ^36^ ^]^

### 3.2 Complexity of Traumatic Wounds

Traumatic wounds that extend deeper than the hypodermis introduce substantial challenges in hemorrhage control, particularly when the injury involves highly vascularized tissues, major blood vessels, or deep anatomical compartments and organs.^[^ ^37^ ^]^ These wounds often cannot be closed by primary intention (Pr‐I), where the skin edges are directly approximated with staples, sutures, or adhesives, as the extent of tissue loss and damage prevents proper alignment of the wound edges.^[^ ^38^ ^]^ Instead, they typically require healing through secondary (Sec‐I) or tertiary intention (Ter‐I), both of which are complex and prolonged processes.^[^ ^39^ ^]^

Specifically, Sec‐I healing occurs when large, deep lacerations necessitate the formation of sufficient granulation tissue to form within the wound bed before epithelialization and then scarring can occur.^[^ ^40^ ^]^ This process is lengthy often yielding substantial scarring; moreover, the regenerated tissue somewhat lacks the structural‐tensile strength compared to the original tissue, rendering it more susceptible to future injury.^[^ ^37^ ^]^ When dehisced wounds are infected or contaminated with foreign objects, Ter‐I is preferred. The injured site is left open and monitored for a specific time window to allow for thorough debridement and infection control. Subsequently, the wound can be surgically closed.

When dealing with deep traumatic wounds, it becomes imperative to understand the interplays between tissue layers. Highly vascularized areas, such as muscles or the scalp, can bleed profusely, while deeper compartments, such as retroperitoneal or thoracic cavities, may conceal hemodynamically significant hemorrhage until life‐threatening hypovolemia occurs.^[^ ^41^ ^]^ Bleeding caused by displaced fractured bones poses significant additional risks, often resulting in severe lacerations of adjacent arterial vessels.^[^ ^42^ ^]^ In such cases, the deployment of compressive hemostatic devices and/or pneumatic tourniquets (in the case of limb injuries) helps mitigate the risk of prolonged bleeding by maintaining sustained pressure, possibly facilitating clot formation.^[^ ^5^ ^]^

Injuries extending beyond the dermis into the hypodermis or deeper layers necessitate vigilant assessment for complications such as compartment syndrome.^[^ ^43^ ^]^ Elevated pressure within confined anatomical spaces, such as the limbs, can exacerbate tissue ischemia and further hinder healing.^[^ ^44^ ^]^ Effective management in these cases requires a comprehensive understanding of anatomical landmarks, including pressure points, vascular territories, and the mechanical effects of the selected HWD. Nonetheless, while the use of HWDs is essential, it is insufficient on its own to arrest catastrophic or “torrential” bleeding without an adhoc delivery mechanism. Recent advancements have focused on the development of drug‐integrated, micro/nanoparticle‐engineered HWDs, as discussed in subsequent sections, to achieve enhanced and more rapid hemostasis at the active bleeding site.

### 3.3 Hemostasis

**Figure 3:**
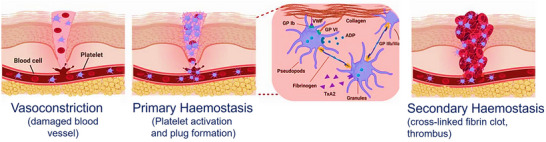
Schematics of the hemostasis process, illustrating three sequential stages of hemostasis involved in stopping bleeding and repairing vascular injury. i) Vasoconstriction: the damaged blood vessel narrows to reduce blood flow and minimize blood loss. ii) Primary hemostasis: platelets adhere to exposed collagen, become activated, and aggregate to form a temporary platelet plug—their activation involves pseudopod extension and the release of signaling molecules such as ADP and TxA2. iii) Secondary hemostasis: the coagulation cascade stabilizes the plug by converting fibrinogen into a fibrin mesh, forming a crosslinked thrombus to seal the injury. Reproduced with permission.^[^ ^46^ ^]^ Copyright 2020, John Wiley and Sons.

Hemostasis begins almost immediately after injury and unfolds over several hours in a three‐stage process (**Figure** **3**).^[^ ^45^, ^46^ ^]^ In the first stage, vasoconstriction is initiated by the release of endothelin from the damaged endothelium, together with catecholamines, prostaglandins, and platelet‐derived growth factor (PDGF) which promote vascular contraction.^[^ ^47^ ^]^ However, these factors alone are insufficient for profuse bleeding control. The second stage, known as primary hemostasis occurs where exposed thrombogenic layers from injured tissue trigger platelet adhesion and activation.^[^ ^48^ ^]^ This involves pseudopod extension and the release of signaling molecules, such as adenosine diphosphate (ADP) and thromboxane A2 (TxA2).

This transformation modifies platelet morphology, enhancing their adhesion and secretion of substances like fibrinogen, fibronectin, von Willebrand factor (vWF), and collagen through tripeptide sequences (arginine–glycine–aspartate (RGD)).^[^ ^49^ ^]^ This facilitates cellular adhesion, and hence the formation of a temporary platelet plug, which is further reinforced during the secondary hemostasis (third stage) phase when the coagulation cascade is synchronously activated leading to fibrin formation and structural re‐enforcement of the platelet plug. The platelet plug becomes enriched with modulatory proteins (hematopoietic cytokines), such as PDGF, pleiotropic transforming growth factor (TGF‐β), epidermal growth factor (EGF), and insulin‐like growth factor (IGF), crucial for subsequent tissue repair.^[^ ^50^ ^]^ These growth factors influence various cell types (fibroblasts, endothelial cells, smooth muscle cells, and monocytes) supporting wound healing. A thrombus thus not only prevents further blood loss but also serves as a scaffold for tissue repair.

Overall, controlling profuse bleeding remains challenging. An overly dense clot or excessive fibrin production can impede oxygen delivery to the wound site, hampering healing and increasing the risk of excessive fibrin coagulum, possibly predisposing the wound to permanent fibrotic scarring and keloids.^[^ ^51^ ^]^ Ideal HWDs should rapidly control bleeding, support balanced fibrin formation, and enable tissue oxygenation.

### 3.4 Ideal Properties of HWDs

**Figure 4:**
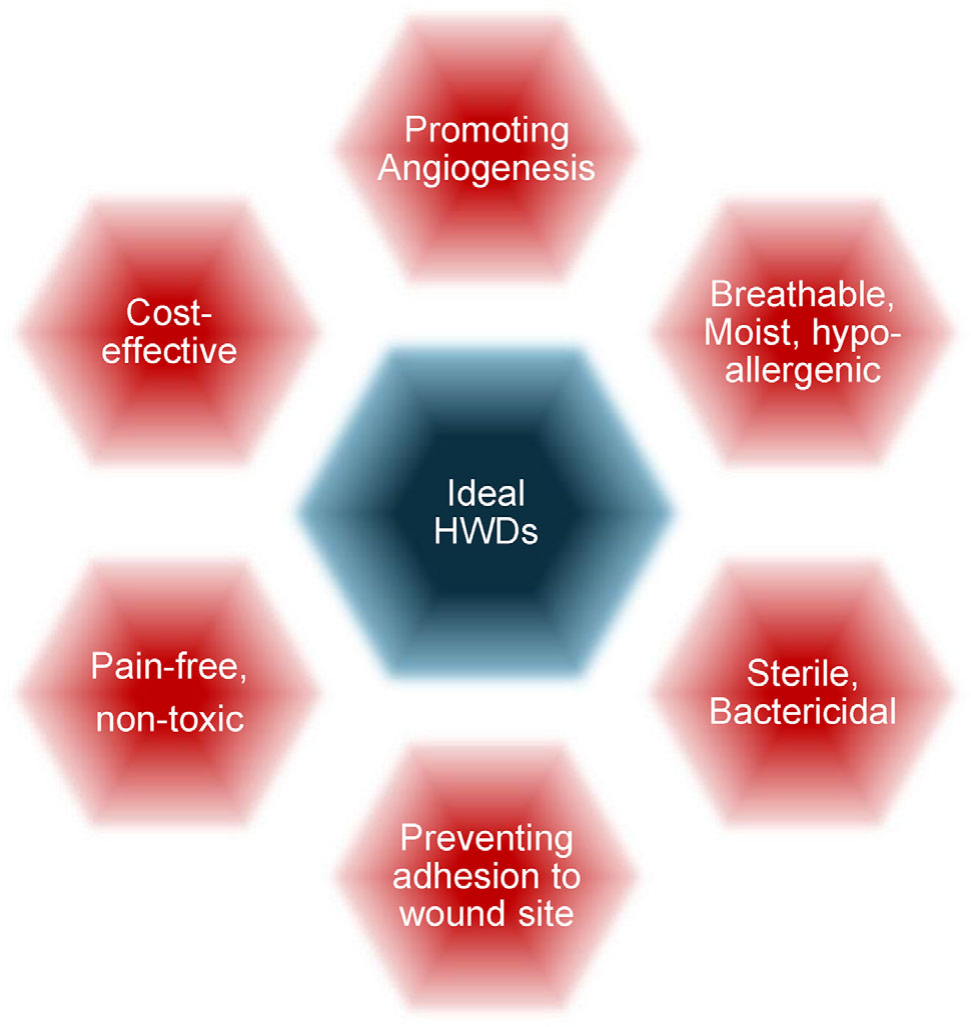
Key properties for the design of ideal hemostatic wound devices (HWDs). Generated using Microsoft Office 365.

Hemostatic wound devices are specialized to stop bleeding and create conditions conducive to sutureless/suture‐assisted healing.^[^ ^6^ ^]^ They should possess a number of properties (**Figure** **4**) to maintain a moist environment, prevent bacterial contamination, allow air exchange (wound breathing), promote neovascularisation (angiogenesis), and support the regeneration of new, clean connective tissue.^[^ ^52^ ^]^ Additionally, they must be sterile, noninvasive, nontoxic, nonallergenic, and cost‐effective at a large manufacturing scale. However, balancing these properties, especially in relation to the broader spectrum of wound types, is a significant challenge.^[^ ^53^ ^]^ HWDs should also minimize pain and prevent wound‐to‐HWD adherence and/or physical interlocking, while exerting the required pressure against the wound for an effective bleeding control. This functional complexity is not met by conventional wound dressings, which are deficient in many of these attributes.

## 4 Landscape of Traditional Hemostatic Dressings

Building on the physiological basis of hemostasis, it is imperative to understand how external interventions can support these natural processes. Similar to how system‐specific microarrays are engineered to overcome biological barriers for targeted, intracellular delivery,^[^ ^54^ ^]^ hemostatic dressings should be designed to interact with complex tissue environments to achieve rapid bleeding control. This section explores the landscape of traditional hemostatic dressings based on their functional mechanisms and limitations.

### 4.1 Passive Dressings

Traditional passive wound dressings, such as sterile gauze and bandages, have been utilized for decades to manage wounds by absorbing exudate (e.g., serosanguineous fluids) and providing protection.^[^ ^55^ ^]^ Woven and nonwoven gauze, including skimmed cotton, viscose, or polyester, exhibit a fibrous composition and are effective at fluid absorption.^[^ ^56^ ^]^ They are commonly applied to clean wounds with minimal exudate, in wet‐to‐dry debridement where the dressing removes necrotic tissue during replacement.^[^ ^57^ ^]^ Although highly affordable, these dressings require frequent changes to prevent complications like maceration and dressing‐to‐wound adhesion, which can cause discomfort and impede healing.^[^ ^58^ ^]^ Cotton‐based materials can adhere to the wound bed or shed fibers, potentially increasing the risk of irritation or bacterial proliferation.^[^ ^59^ ^]^

### 4.2 Active Dressings

Alginate‐based dressings exhibit a slight hemostatic effect due to their high calcium/sodium ion content.^[^ ^60^ ^]^ Commonly available alginate dressing products include Biatain Alginate (Coloplast, Denmark, EU) and Tegaderm (3M, US).

For example, Biatain Alginate is a highly absorbent dressing, made of 85% alginate and 15% carboxymethylcellulose (CMC), designed for moderate to heavily exuding wounds of various sizes and depths.^[^ ^61^ ^]^ It is effective for managing exudate, promoting wound (e.g., venous/arterial and diabetic foot ulcers) compression and subsequent healing. Additionally, the release of Ca^2+^ helps control minor bleeding in superficial wounds. However, it is not recommended for controlling heavy bleeding. A key limitation is its reduced efficacy in wounds with minimal exudate, as the dressing relies on moisture to activate.

Similarly, 3M Tegaderm High Integrity Alginate Dressing is a highly absorbent primary dressing designed for moderate to heavily exuding wounds.^[^ ^62^ ^]^ Like Biatain Alginate, it absorbs wound exudate and forms a gel, creating a moist environment that promotes healing. The dressing also supports autolytic debridement and can help manage minor bleeding. A key advantage of Tegaderm is its high structural integrity, making it easier to remove without leaving residue in the wound bed. However, it requires a secondary dressing for secure application and optimal performance, adding complexity and cost, which can be challenging in resource‐limited settings. While both dressings are effective for managing actively exuding wounds, neither is suitable for dry wounds or heavy bleeding.

**Figure 5:**
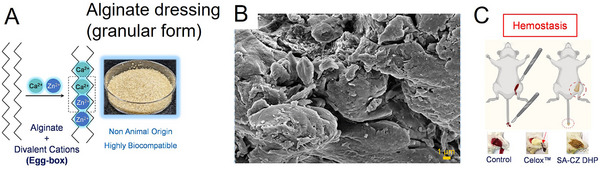
A) Schematic representation of the “egg‐box” scaffold structure formed by alginate in the presence of Ca^2+^ and Zn^2+^ ions. B) Scanning electron microscopy (SEM) image of Ca^2+^/Zn^2+^‐enriched alginate powder dressings. C) Tail tip amputation and femoral artery incision models in Sprague–Dawley rats (≈200 g), comparing the hemostatic performance of control, commercial Celox, and granular Ca^2+^/Zn^2+^‐enriched alginate dressings. Reproduced with permission.^[^ ^66^ ^]^ Copyright 2024, American Chemical Society.

Academic research is exploring new avenues to circumvent these limitations. Researchers are focusing on understanding the physicochemical pathways and interactions between blood components and metal ions (Ca^2+^ and Zn^2+^) to drastically accelerate hemostasis. Upon contact with a wound, calcium ions in alginate dressings are exchanged with sodium ions from the blood, building a defensive film that aids in controlling bleeding.^[^ ^30^ ^]^ This ion exchange may actively facilitate the formation of a fibrin clot. Ponsen et al.^[^ ^63^ ^]^ fabricated a zinc‐supplemented calcium alginate compress, capable of providing positive hemo‐ionic interactions by releasing both Zn^2+^ and Ca^2+^ ions upon contact with blood. Its styptic efficacy and blood clot stability were demonstrated, with in vivo results suggesting enhanced angiogenesis and accelerated epithelial cell maturation.^[^ ^63^ ^]^ The enzymatic factors leading to blood clotting and fibrin formation require Ca^2+^ ions, whereas Zn^2+^ increases platelet aggregation.^[^ ^64^ ^]^ Similarly, Kumar et al.^[^ ^65^ ^]^ developed a granular Zn^2+^/Ca^2+^‐enriched alginate hydrogel as a hemostatic tool. Upon release of the metal ions, the formulation recedes to its original hydrogel state (i.e., sodium alginate), enabling facile and atraumatic removal from the wound, causing no further bleeding (**Figure** **5**).^[^ ^65^ ^]^ The hydrogels demonstrate excellent biocompatibility, as evidenced by their minimal impact on red blood cells and low in vitro toxicity.^[^ ^66^ ^]^ Importantly, the formulation is nonanimal sourced, ensuring higher safety and nonimmunogenicity, which enhances its suitability for widespread clinical use. Despite these advantages, these hydrogels heavily rely on ion exchange for coagulation.^[^ ^66^ ^]^ This may limit their effectiveness in certain physiological conditions, possibly requiring frequent mechanical replacement of the bandage, hence disrupting wound healing. Further studies should be conducted to assess their long‐term safety and efficacy.

Additional efforts to improve traditional alginate dressings have introduced some functional enhancements, such as antibacterial activity.^[^ ^67^ ^]^ For instance, bismuth tribromophenate has been effectively impregnated into fine‐mesh petrolatum dressing patches (Xeroform), reducing the risk of infection and promoting epithelialisation. Recently, this system has been repurposed as an inexpensive and effective alternative to synthetic skin grafts, which was found to promote the development of healthy granulation tissue when employed to wrap Mohs pedicle flaps.^[^ ^68^ ^]^

Overall, alginate‐based dressings demand frequent changing and are not suitable for tackling significant bleeding and/or wounds with dehiscence or necrotic tissues.^[^ ^30^ ^]^ These dressings are highly effective for wounds with heavily oozing exudate, such as ulcer wounds, due to their strong absorption capacity. Future work should be directed at engineering dressings with self‐activated clot‐promoting properties to yield hemostatic devices capable of managing actively bleeding sites marking a breakthrough for clinical applications.

### 4.3 Advanced Dressings

Unlike traditional materials, advanced dressings often integrate multiple biofunctionalities, such as increased specific surface, responsiveness to stimuli, in situ drug delivery, and antimicrobial activity through hybrid compositions of biological/nonbiological components.

Chitosan, a naturally derived cationic biopolymer (p*K*_a_ ≈ 6), has gained attention for its potentially hemostatic properties.^[^ ^69^ ^]^ This is due to its ability to interact with biological tissues and blood components. Its positive charge promotes adhesion to negatively charged glycosylated cell membranes, likely facilitating clot formation and enhancing tissue adhesion through electrostatic interactions.^[^ ^70^ ^]^ Further modifications, such as grafting cationic quaternary ammonium groups onto its backbone, improve its adhesive properties, making it effective for wound sealing.^[^ ^71^ ^]^ Additionally, chitosan activates platelets and interacts with blood cells to support the coagulation cascade.^[^ ^72^ ^]^ Similarly, ε‐polylysine, another cationic biopolymer (p*K*_a_ ≈ 9), may also contribute to hemostasis by enhancing adhesion to oppositely charged biological surfaces.^[^ ^73^ ^]^ While both biopolymers hold promise for effective biological adhesion at physiological pH, possibly supporting the formation of a stable clot, they may suffer delayed activation, limiting overall effectiveness in situations requiring rapid or substantial clot formation.

In a 2009 patent by Van Den Berg and Hoekstra^[^ ^74^ ^]^ (US 2009/0304780 A1), the authors introduced a novel wound dressing integrating buckwheat honey with a metal ion–citric acid carrier, marking a shift toward nonpassive, bioactive advanced solutions. This approach leverages the natural acidity and antimicrobial properties of buckwheat honey to possibly promote fibroblast proliferation and reduce inflammation, key indicators of enhanced wound healing. The patent reflects growing interest in combining naturally sourced ingredients with advanced delivery systems to create multifunctional hemostatic dressings that actively modulate the wound microenvironment. In a 2017 patent (US9782512B2) filed by Von Blücher et al.^[^ ^75^ ^]^, a multilayered wound dressing, combining bioactive collagen‐based hydrocolloid and spherical activated microporous carbon (high surface area), was introduced, offering both therapeutic and adsorptive capabilities. This design manages wound exudate, helping neutralize toxins, and supports tissue regeneration, representing a shift from passive to advanced multifunctional wound care solutions. This innovation underscores the broader trend toward advanced dressings that blend naturally derived materials with engineered functionalities for enhanced clinical performance.

## 5 HWDs with Integrated Particle Technology

Microparticle‐integrated and nanoengineered systems offer unprecedented control over the spatial and temporal delivery of hemostatic agents, enabling rapid, localized coagulation where it is needed. Unlike bulk materials, these platforms can be functionalized with procoagulant, antimicrobial, and/or anti‐inflammatory agents, enhancing therapeutic efficacy while minimizing systemic side effects. Their high surface‐area‐to‐volume ratio allows for faster interaction with blood components, accelerating clot formation under extreme conditions.^[^ ^10^ ^]^ These systems represent a paradigmatic shift in trauma care, moving beyond passive materials toward “intelligent,” bioresponsive platforms. Despite these advantages, challenges in scalability, regulatory approval, and long‐term biocompatibility persist. Moreover, potential risks, such as immunogenicity or concerns related to long‐term biodegradability, warrant careful evaluation. Without addressing these limitations, the clinical translatability and safety profile of such advanced systems remain uncertain.

### 5.1 Zeolite‐Based Dressings

Zeolite‐based styptic agents, marketed under the name QuikClot, have shown promise in controlling severe bleeding.^[^ ^76^ ^]^ Their porous aluminosilicate structure, which contains Na^+^, Ca^2+^, Mg^2+^, Al^3+^, and silicon oxides, facilitates this coagulation process by adsorbing water molecules from bleeding‐active wounds, while concentrating clotting factors and platelets.^[^ ^30^ ^]^ Animal studies have confirmed their effectiveness, including microbicidal activity, without causing cytotoxicity, zoonosis, or allergen‐triggered reactions.^[^ ^30^, ^77^ ^]^

Recently, Zhu et al.^[^ ^78^ ^]^ combined *Bletilla striata* polysaccharides and ZSM‐5 zeolite into a composite wound dressing.^[^ ^78^ ^]^ ZSM‐5 led to larger hemostatic efficiency, enhanced antibacterial activity, facilitating wound healing. At the optimal mass polysaccharides‐to‐zeolite ratio (2:1), both in vivo and in vitro tests showed that the minimum clotting time was 41 ± 5 s, compared to the control group (343 ± 22 s). This was likely yielded through zeolite‐driven extrinsic coagulation pathways, due to the high porosity and superior water uptake capacity, accompanied by minimal cytotoxicity.

However, the applicability of zeolites is hindered by several issues. The exothermic reaction that occurs upon contact with blood can raise the wound temperature up to 75–78 °C, potentially causing thermal shock and subsequent injury.^[^ ^79^ ^]^ Additionally, zeolite particulates can be difficult to remove from wounds, which may lead to irritation, foreign body reactions, or bacterial infection. Furthermore, in high‐pressure wounds with copious bleeding, the free mobility of the particles, with a typical size of 40–60 µm can dramatically reduce their effectiveness.^[^ ^80^ ^]^

**Figure 6:**
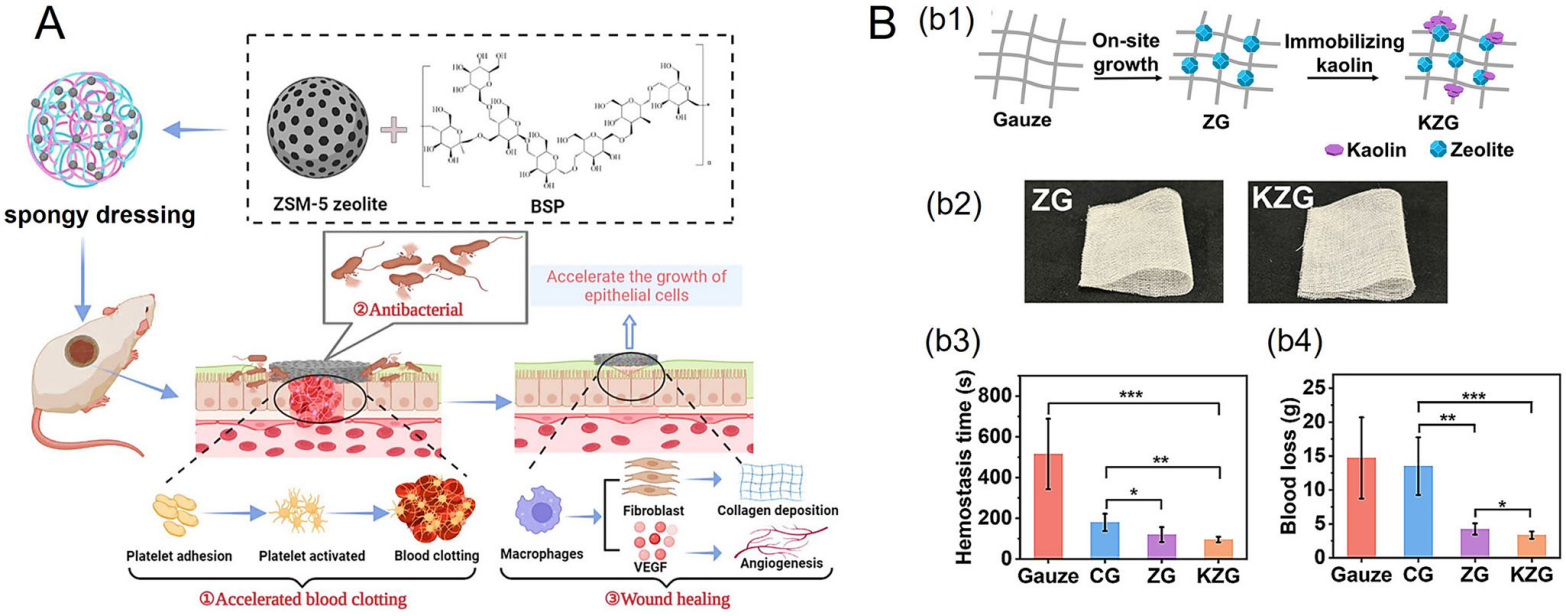
A) Schematic illustration of the application mechanism of spongy dressings incorporating ZSM‐5 and *Bletilla striata* polysaccharide (BSP) for wound healing, highlighting key stages of the healing process. Reproduced with permission.^[^ ^78^ ^]^ Copyright 2023, Springer Nature. B) Schematic representation of the preparation of b1) kaolin zeolite gauzes (KZG) through postmodification of a commercial zeolite hemostatic gauze; b2) optical images of unmodified zeolite gauze (ZG) and kaolin zeolite gauze (no scale bar provided). Quantitative analysis of b3) hemostatic time and b4) blood loss in a rabbit femoral artery injury model. Reproduced with permission.^[^ ^76^ ^]^ Copyright 2024, American Chemical Society.

Kaolin, a clay mineral, offers an alternative hemostatic solution with a number of advantages. It does not induce exothermic reactions, eliminating the risk of thermal injury.^[^ ^76^ ^]^ Kaolin powder has been successfully incorporated into QuikClot Combat HWDs and commercialized, yielding a less/nongranulated structure.^[^ ^81^ ^]^ This has ensured that no residual particles may remain in the wound, minimizing the risk of later inflammation and infections. Additionally, kaolin maintains consistent clotting efficiency even when hydrated (**Figure** **6**), unlike zeolite whose effectiveness significantly decreases in its hydrated form.^[^ ^76^ ^]^ Kaolin achieves hemostasis by activating Factor XII (FXII), initiating the intrinsic clotting cascade that leads to generation of thrombin and thereby fibrin formation, and providing a hemostatic response with over remarkable efficiency.^[^ ^3^ ^]^ However, a potential limitation arises in patients with trauma‐induced coagulopathy, where thrombin generation is impeded downstream of Factor XII activation.^[^ ^30^ ^]^ Despite these challenges, kaolin‐based dressings hold significant promise for enhancing hemostasis in trauma care, offering a safe and reliable alternative to zeolite.

### 5.2 Microcomposites

Composite particles have gained attention for their efficacy in hemorrhage control and wound healing due to their multifunctional properties.^[^ ^82^ ^]^ Wang et al.^[^ ^83^ ^]^ incorporated sodium polyacrylate particles (150–380 µm) into a packet‐form styptic dressing. The material demonstrated rapid absorption of blood, cellular aggregation, accelerating coagulation. In a groin‐penetrating trauma swine model hemostasis was achieved, with a significantly faster application time (19.0 ± 4.6 s) compared to Celox (169.0 ± 73.5 s) and standard gauzes (187.8 ± 1.7 s).^[^ ^83^ ^]^ Unlike traditional dressings, this formulation required no external compression, simplifying deployment and removal.^[^ ^84^ ^]^ Despite its promising performance, testing across diverse trauma models and assessment of long‐term biocompatibility should be explored.

**Figure 7:**
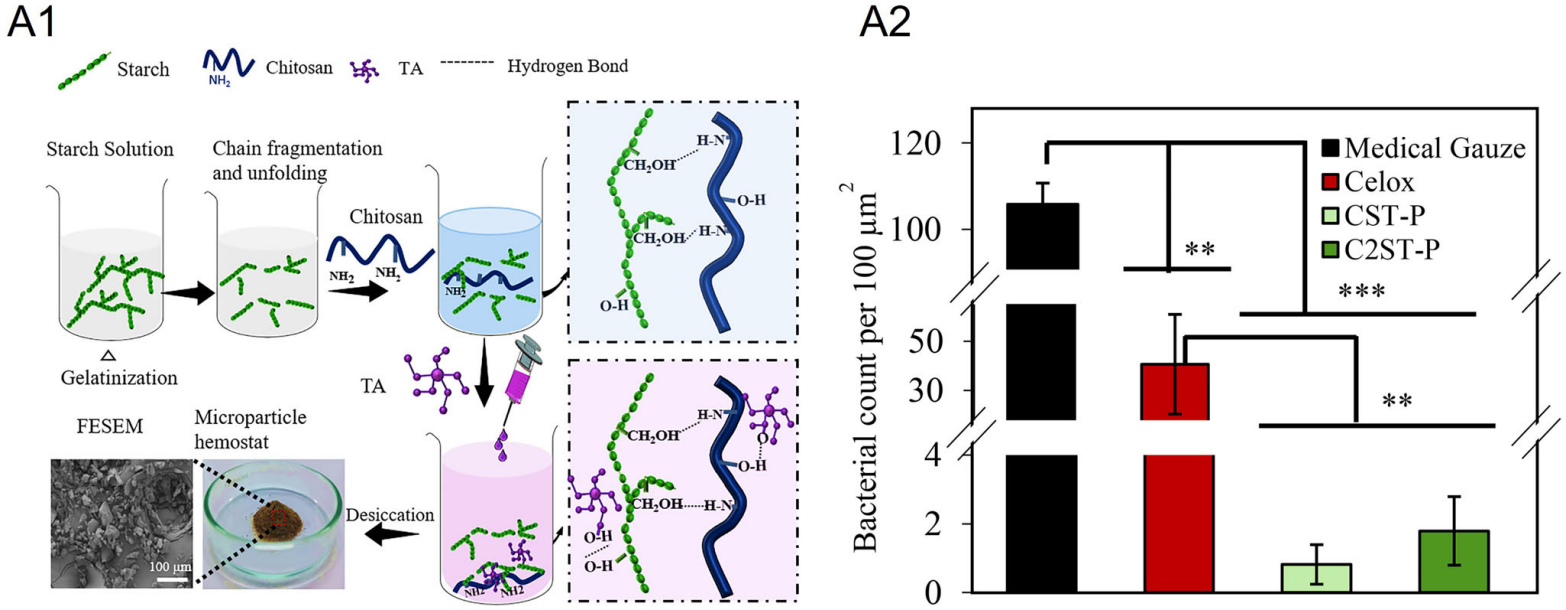
A) Schematic illustration of the synthesis process and proposed chemical pathway between biopolymers and polyphenol (chitosan, starch, and tannic acid) in the hemostatic formulations. B) Bacterial response to control (medical gauze), Celox, and hemostatic microparticles CST‐P (1% chitosan/TA/potato starch) and C2ST‐P (2% chitosan/TA/potato starch) after direct contact with Gram‐negative *P. mirabilis*. The graph shows the number of live bacteria per 100 µm^2^ on each sample. Reproduced with permission.^[^ ^85^ ^]^ Copyright 2024, Elsevier.

Tithy et al.^[^ ^85^ ^]^ developed chitosan‐based microparticle hemostats, crosslinked with tannic acid (TA), incorporating hydrothermally treated starch.^[^ ^85^ ^]^ These particles, highly polydisperse in size (5–50+ µm), offered rapid clotting response, with bleeding times reduced to 13 ± 4 s in mouse liver lacerations and ≈40 s in rat tail amputations, comparable to commercial Celox. The addition of starch improved erythrocyte compatibility, promoting broad‐spectrum bactericidal efficacy (>90% for *Staphylococcus aureus*, >80% for *Escherichia coli*, >80% for *Proteus mirabilis*), achieving a wound‐healing closure of ≈94% (**Figure** **7**). However, challenges such as particle size uniformity and scalability for large mammal hemorrhage control remain to be addressed.

Free‐flowing microgel composites (100–500 µm) made of carboxymethyl starch–cellulose, represent a novel approach to hemostasis and wound healing.^[^ ^86^ ^]^ Their rapid swelling and crosslinking upon contact with exudate enable the formation of an integrated composite microgel that conforms to irregular wound bed geometries, providing a moist environment crucial for tissue regeneration.^[^ ^87^ ^]^ Enhanced by sustained silver nanoparticle (AgNP) release, these microgels exhibited antimicrobial properties, yet their long‐term performance in highly exuding wounds and potential cytotoxicity^[^ ^88^ ^]^ warrant further investigation.

A novel self‐gelatinizing hemostatic powder, made of CMC calcium (CMC‐Ca) and lysine, was proposed by Zhao et al.^[^ ^89^ ^]^ It exhibited excellent water uptake capacity (up to 30 times of its weight) and rapid gelation (within ≈4 s). The powder, composed of mostly elongated filaments (≈100 µm) achieved clot formation in under 3 min. Nearly complete tissue recovery was attained within 14 days when applied to injured mouse dermis.

Similarly, Zhao et al.^[^ ^90^ ^]^ showcased a self‐gelling, wet adhesive powder system for rapid hemostasis, capable of stopping bleeding in 10 s and reducing blood loss by more than sixfold in murine models of femoral artery and liver injury.^[^ ^90^ ^]^ The formulation relied on specific formulations, namely (polyethylenimine/poly(dimethylammonium chloride)/(poly(acrylic acid)/poly(sodiumstyrenesulfonate)/alkylated chitosan)), yielding high mechanical properties (storage modulus *G*′ ≈ 64 kPa) and promising stretchability. With no treatment, the clotting time of whole blood (control) was ≈400 s, which was reduced to ≈160 s in the presence of the self‐gelling powders. However, concerns due to complex interactions between the nanoparticles and polymer matrices may arise, and more comprehensive in vivo studies may help confirm both efficacy and safety across diverse wound types.

Interestingly, Nagrath et al.^[^ ^91^ ^]^ proposed mesoporous tantalum‐loaded bioactive glass fibers (Ta‐MBG) that may offer a stable matrix for clot formation.^[^ ^91^ ^]^ Unlike powdered formulations, these micrometer‐scaled electrospun fibers exhibited lower ion release and highly negative surface charges, potentially enhancing their stability in wet wound environments. Ta‐MBG improved clotting compared to untreated controls and the commercial hemostatic agents (Surgicel SNoW), with the highest coagulation performance observed at a tantalum concentration of 1 mol%. Furthermore, Ta‐MBG demonstrated no cytotoxic effects on rodent fibroblasts, suggesting good biocompatibility. However, the study does not elucidate the role of tantalum in enhancing the intrinsic coagulation cascade or wound healing. Further investigation is needed to understand both underlying mechanisms and chemical pathways, in order to assess the clinical applicability of these materials. Key microcomposite systems are presented in **Table** **1**.

**Table 1:** HWDs engineered with micro‐/nanocomposites based on their physical and performance properties.

Method	Type	Composition	Size	Properties	Reference
Micrometet scale	Microparticles incorporated into dressings	Sodium polyacrylate	150–380 µm	Hemostasis (groin‐penetrating trauma swine model) within 19.0 ± 4.6 s vs standard gauzes (187.8 ± 1.7 s); promoted cellular aggregation; accelerated coagulation	[83]
	Microparticle‐assisted hemostats, crosslinked with tannic acid (TA), incorporating hydrothermally treated starch	Chitosan‐based, starch	5–50+ µm	Bleeding times reduced to 13 ± 4 s (mouse liver laceration) and ≈40 s (rat tail amputations); Broad‐spectrum bactericidal activity (>90% for *S. aureus* and >80% for *E. coli*); Wound‐healing closure of ≈94%.	[85]
	Free‐flowing, self‐crosslinking microgels	Carboxymethyl starch–cellulose	100–500 µm	Forming integrated hydrogel upon contact with exudate to conform to irregular wound bed geometries; supporting cell growth; open wound (<10%) compared to the corresponding control (≈ 30%) after 14 days; antimicrobial properties	[86]
	Self‐gelling hemostatic composite powder	Carboxymethylcellulose calcium, lysine	≈100 µm (elongated filaments)	Excellent water uptake (up to 30 times its weight), rapid gelation (within ≈4 s); clot formation within 3 min; mouse skin trauma model: nearly complete tissue recovery within 14 days.	[89]
	Self‐gelling, wet, adhesive powdered system	Polyethylenimine/poly(dimethylammonium chloride)/(poly(acrylic acid)/poly(sodium styrenesulfonate)/alkylated chitosan	>50 µm	Forming an in situ hydrogel patch within 3 s upon contacting exudated fluids; clotting time reduced to ≈160 s compared to control (whole blood ≈ 400 s); reducing blood loss by more than sixfold	[90]
Nanoscale	Solid‐state sintered composites	Sepiolite–silver and metal oxides	≈20–30 nm (fiber diameter) Ag NPs (≈8 nm)	Fibrous morphology; at 5% Ag content, bactericidal efficacy almost 100% against pathogens (*E. coli* and *S. aureus*); biocompatibility	[92]
	Composites	Sepiolite–silver nanoparticulate	13–18 nm (pore diameter) Ag NPs ≈9.9 nm	Remarkable hemostatic properties (no exothermic reaction unlike zeolites); in tail vein hemorrhage mouse model, arrested bleeding within 103 ± 8 s; significant in vivo healing response; inhibited bacterial growth	[93]
	Nanonets	Hydrogel‐based (Activated Coagulation Factor Products (ACFP))	≈25 nm (estimated)	Peptide‐based hydrogel inspired by human defensin (HD6); rat femoral vein transection: reduced blood loss (<0.8 g) compared to control (≈3 g); accelerated hemostasis within ≈20 s (control: >80 s); antimicrobial activity.	[94]
	Nanofibers loaded with *Capparis sepiaria* plant extract	Sodium alginate, polyvinyl alcohol (PVA), poly‐(d,l‐lactide)‐*co*‐glycolide (PLGA)	50–100 nm (estimated fibre diameter)	Accelerated blood clotting (absorbance values <0.25; control >0.5); bactericidal activity against *P. aeruginosa*, *E. coli*, *and S. aureus*, low cytotoxicity (cell viability over 90%)	[96]
	Composites co‐loaded with ZnO nanoparticles and a combination of fluoroquinolone antibiotics	Sodium alginate, poloxamer 407, mastic gum	ZnO NPs (≈39 nm)	ZnO NPs contributed to reducing blood clotting index (halved absorbance values compared to control (≈0.5)); antimicrobial activity	[97]
	Nanofibrous composites featuring a zinc‐based metal–organic framework (MOF)	Casein–polyvinyl alcohol	80–150 nm	Highly hydrophilic; antibacterial properties against Gram‐positive/negative; Zinc positively interacted with red blood cells, halving clotting times from over 400 s (control) to ≈200 s	[98]
	Biogenic nanostructures	Polyvinyl alcohol–hypromellose and silver nanoparticles, in the presence of xanthan gum or sodium carboxymethyl cellulose	≈25–49 nm (nanosized particles)	Accelerated blood clotting; broad spectrum in vivo antibacterial activity; significant reduction in wound area after 31 days (composites: <2 mm^2^; control ≈ 7 mm^2^ (estimated))	[100]
	Nanocrystals	Polydimethylsiloxane and hydrophobic‐modified cellulose, TA, decylamine (DA)	≈207 ± 20 nm 245 ± 35 nm (with TA) 346 ± 30 nm (with DA).	Reduced blood loss by over 90%; shortened clotting time by over 75%; minimized bacterial adhesion by ≈95%; achieving water contact angle of ≈160°	[101]

### 5.3 Nanocomposites

Sepiolite‐based nanocomposites, modified with nanosized silver, show great promise for wound healing and hemostasis.^[^ ^92^ ^]^ Their fibrous morphology, even after sintering at temperatures below 500 °C, is preserved, promoting skin compatibility and water retention. With as low as 5% wt Ag° content, dramatic bactericidal efficacy (≈100%) against pathogens (*E. coli* and *S. aureus*) was achieved. Similar sepiolite‐AgNP composites have been produced by Jiang et al.^[^ ^93^ ^]^ Elemental Ag° NPs were attained by UV‐assisted irradiation of Ag^+^ ions (20 mmol of AgNO_3_ as the precursor), yielding remarkable bactericidal efficacy. In tail vein hemorrhage mouse model, the nanocomposites arrested bleeding within 103 ± 8 s and inhibited bacterial growth. Unlike zeolites, sepiolite nanocomposites trigger no exothermic reactions; notwithstanding, further research is needed to confirm their effectiveness and safety, particularly in clinical settings.

Yuan et al.^[^ ^94^ ^]^ recently developed de novo biomimetic, peptide‐based, dynamic hydrogels (Activated Coagulation Factor Products) inspired by human α‐defensin 6 (HD6).^[^ ^94^ ^]^ These systems offered multifunctionalized solutions combining accelerated hemostasis and antimicrobial activity, thereby facilitating wound healing. They were engineered to mimic host‐defence peptides (HD6) nanonet structures, which proved biocompatible and effective at promoting tissue repair while preventing irreversible wound‐to‐tissue adhesion. In rat femoral vein transection, Activated Coagulation Factor Products (ACFP) yielded reduced blood loss (<0.8 g) compared to its control (≈3 g), and accelerated hemostasis within ≈20 s (control: >80 s).^[^ ^95^ ^]^ However, some challenges remain, including optimizing its physicochemical properties for broader spectrum applications, understanding its wound‐healing mechanisms, and validating efficacy through in vivo studies.

Another promising methodology was presented by Ndlovu et al.,^[^ ^96^ ^]^ proposing sodium alginate‐based nanofibers loaded with plant extract (*Capparis sepiaria*) for rapid hemostasis and wound healing.^[^ ^96^ ^]^ In the presence of nanofibers, a higher blood clotting rate was attained compared to the whole blood control. Moreover, the nanofibers promoted large bactericidal activity against *Pseudomonas aeruginosa*, *E. coli*, and *S. aureus*, low cytotoxicity (cell viability over 90%; incubation time >50 h), and enhanced cell migration, as evidenced by in vitro scratch wound‐healing assays. Promising antibacterial and blood clotting properties were reported by the same research group when fabricating similar, dissolvable, composite dressings co‐loaded with zinc oxide (ZnO) nanoparticles and a combination of fluoroquinolone antibiotics, such as ciprofloxacin.^[^ ^97^ ^]^ Despite these strengths, the need for comprehensive mechanistic studies to fully understand the extract–polymer/nanoparticle‐polymer synergy, compared to commercial gel‐based wound dressings, should be conducted to ensure their broader spectrum efficacy in clinical applications where remarkable mechanical strength is required.

**Figure 8:**
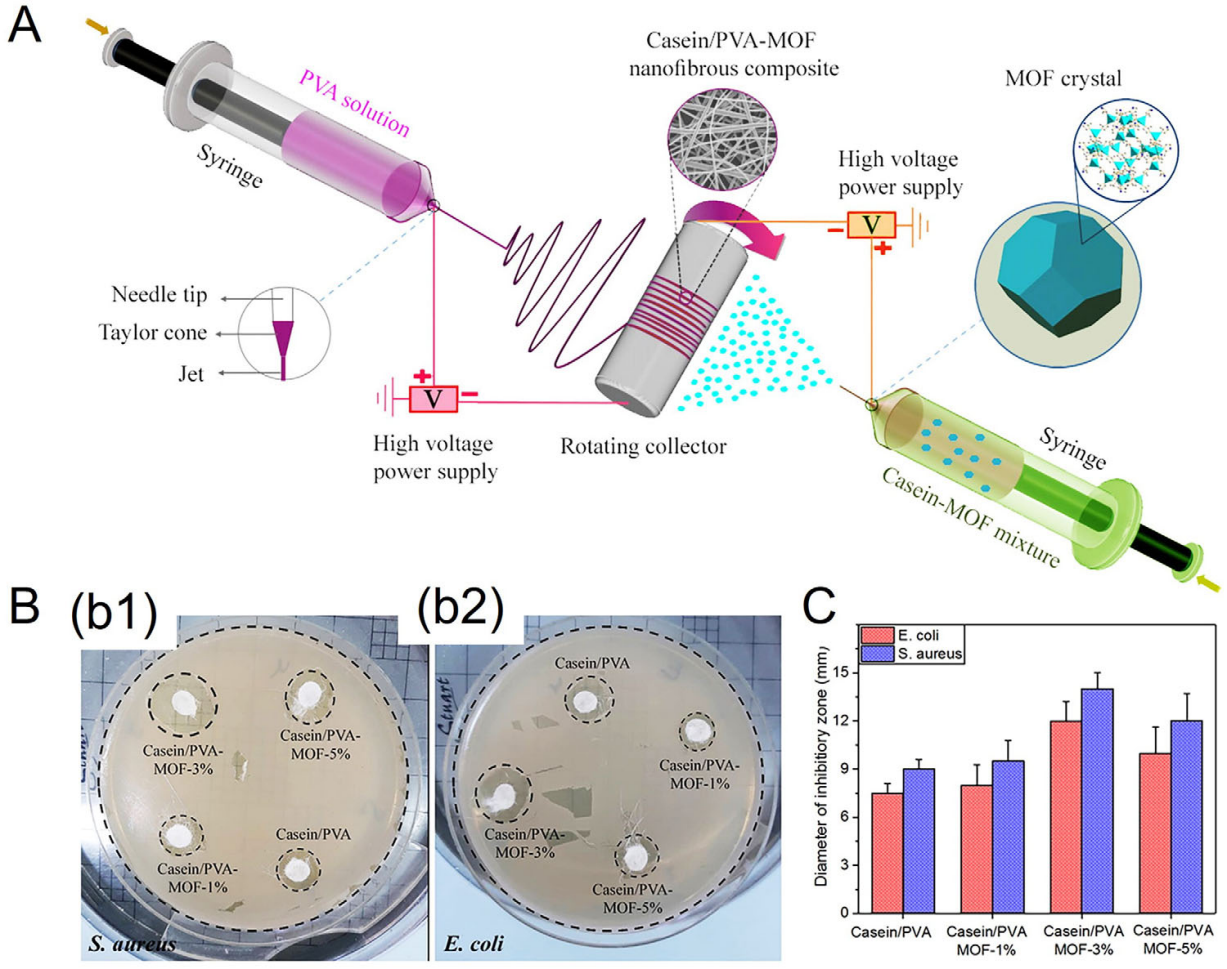
A) Schematic depiction of concurrent electrospinning for the fabrication of casein/PVA‐MOF nanofibrous composites. B) Antibacterial activity of the nanofibrous composites, with inhibition zone photographs against b1) *S. aureus* and b2) *E. coli*. C) Quantitative measurement of the inhibition zone diameter for casein/PVA‐MOF nanofibrous composites against *S. aureus* and *E. coli*. Reproduced with permission.^[^ ^98^ ^]^ Copyright 2023, Taylor & Francis.

Innovatively, hydrophilic casein–polyvinyl alcohol (PVA) nanofibrous composites (80–150 nm) featuring a zinc‐based metal–organic framework (MOF) have been developed with strong hemostatic and Gram‐positive/negative antibacterial properties (**Figure** **8**).^[^ ^98^ ^]^ Zinc positively interacted with red blood cells, almost halving the clotting times from over 400 s, down to ≈200 s. Although the tensile strength (5.2 MPa) of these nanofibrous at the elongation break (strain ≈ 35%) was promising, challenges may include impaired mechanical properties when lower MOF concentrations are used. In respect of the potential cytotoxicity associated with zinc‐based MOF, Zn showed system‐specific, conditionally cytotoxic effects, depending on the material type and cell line. For instance, endothelial cells may exhibit larger tolerance to Zn‐based biodegradable MOFs than other tested cells.^[^ ^99^ ^]^

Moreover, new avenues are being explored in the development of dual functional hemostatic and post‐hemostatic wound dressings. Singh et al.^[^ ^100^ ^]^ fabricated biogenic nanostructures utilizing polyvinyl alcohol–hypromellose and silver nanoparticles, in the presence of xanthan gum or sodium carboxymethyl cellulose. These dressings showed accelerated blood clotting, supporting sustained wound healing and reducing post‐traumatic inflammation. This was likely due to intrinsic pathways of blood coagulation driven by elemental silver in addition to provision of broad spectrum in vivo antibacterial activity. Although silver nanoparticles can be significantly cytotoxic, biocompatibility tests on dermal and immune cells (macrophages, human keratinocytes (HaCaT), and fibroblasts (L929)) revealed that those with a larger size (300–800 nm) exhibited higher biocompatibility, yielding over 80% cell viability.^[^ ^100^ ^]^

An ultra‐hydrophobic (water contact angle of ≈160°) gauze engineered with nanocomposites was developed by Chen et al.^[^ ^101^ ^]^ Its surface was loaded with polydimethylsiloxane and hydrophobic‐modified cellulose nanocrystals, reducing blood loss by over 90%, shortening clotting time by more than 75%, and minimizing bacterial adhesion by more than 95%. The gauze promotes rapid hemostasis by activating coagulation factors, fibrin formation, and platelet activation, while retaining lightweight, breathable, and nonallergenic properties. However, performance tests in broader bleeding scenarios remain for the future. Key nanocomposite systems are presented in Table 1.

### 5.4 Nanoparticles

Nanoparticles offer potential for hemostasis and the reduction of postoperative complications such as bacterial infection and delayed wound healing.^[^ ^102^ ^]^ Central to their efficacy is their high specific surface area (SSA), which allows NPs to adsorb large amounts of coagulation proteins, growth factors, and therapeutic agents.^[^ ^103^ ^]^ Therefore, smaller nanoparticles (≈50 nm) exhibit stronger procoagulant effects compared to larger particles (>100 nm).^[^ ^103^, ^104^ ^]^ NPs can act as scaffolds, concentrating and activating clotting factors, with advantageous wound penetration that allows for more site‐specific delivery of therapeutic agents.^[^ ^103^, ^105^ ^]^

The electrostatic interaction of NPs with platelets and/or endothelial receptors is another critical mechanism.^[^ ^106^ ^]^ Some nanoparticles are indeed capable of activating FXII, which contains positively charged patches, through contact with negatively charged surfaces, such as silica, amplifying the intrinsic pathway towards coagulation and thrombogenicity.^[^ ^107^ ^]^

In contrast, positively charged NPs can enhance platelet aggregation by forming crossbridges with negatively charged sialic acid residues on platelet surfaces, likely establishing a type of electrosteric mechanism of NP–platelet stabilization.^[^ ^103^, ^108^ ^]^

In recent in vitro blood clotting experiments using whole swine blood, it was shown that increasing the concentration of keratin–catechin nanoparticles (KE‐NPs) in cellulose hydrogels would result in the formation of larger blood clots.^[^ ^109^ ^]^ Furthermore, in vivo assays, using rat models of liver puncture and tail amputation, revealed that the cellulose–KE–NPs combination was more effective than using either cellulose hydrogel or the NP alone, as it synergistically accelerated hemostasis and reduced bleeding.^[^ ^46^ ^]^

Later, Chen et al.^[^ ^110^ ^]^ crafted an innovative NP‐in‐microgel (NP‐µGel) system, with promising styptic properties, by incorporating MnO_2_ NPs (71 ± 7 nm) into gelatine microspheres (5–60 µm), crosslinked with polydopamine.^[^ ^110^ ^]^ This structure exhibited a stronger blood coagulation activity, as shown by its lower blood‐clotting index (25.3%) and in both in vitro and in vivo models, compared to the NP‐free µGel (31.8%) and its corresponding control (100%).

Future efforts should focus on refining NP‐integrated HWD materials to enhance clotting performance, minimize adverse reactions and cytotoxicity, and incorporate additional functionalities, making them versatile tools for advanced surgical and trauma care.

### 5.5 Microcapsule‐Assisted HWDs

Biopolysaccharide‐based nano‐/microcapsules demonstrate significant promise in biomedical applications, including wound dressings, due to their customizable size, shape, surface, and bioactive properties, as well as their capacity for controlled release and biocompatibility.^[^ ^111^ ^]^ These microcapsules not only accelerate tissue regeneration but also contribute to hemostasis, which is the most critical stage of wound repair.^[^ ^112^ ^]^

**Figure 9:**
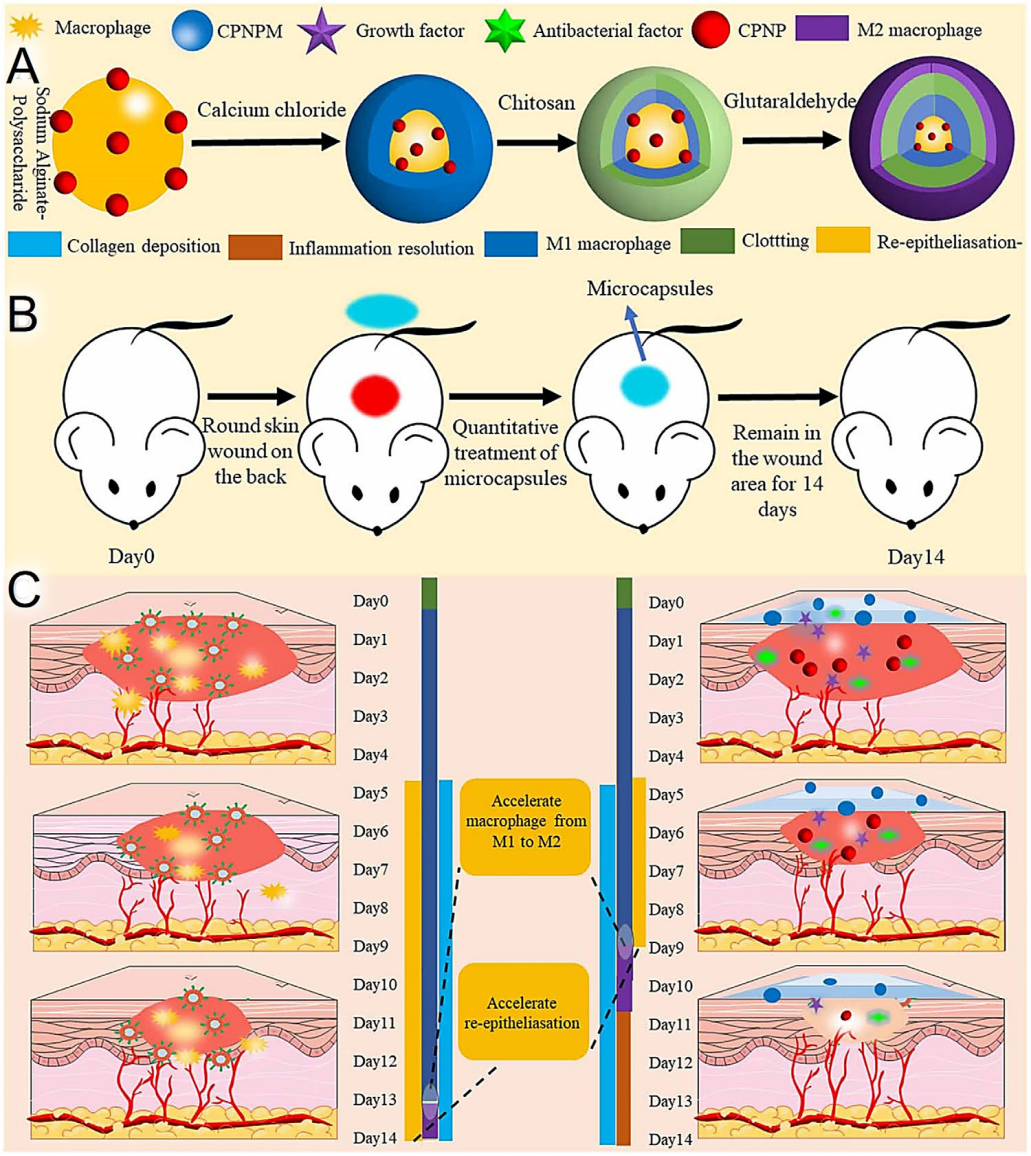
Schematic illustration of the skin wound‐healing process with/without microcapsules, highlighting their preparation and application. A) Fabrication of layer‐by‐layer self‐assembled microcapsules. B) Application of microcapsules in skin wound treatment. C) Comparative stages of skin wound healing with and without microcapsule intervention. Reproduced with permission.^[^ ^113^ ^]^ Copyright 2022, IOP Publishing, Ltd.

For instance, Wang et al.^[^ ^113^ ^]^ fabricated *Codonopsis pilosula* polysaccharide‐based microcapsules for HWDs via a layer‐by‐layer self‐assembly method using sodium alginate, calcium chloride, and chitosan (**Figure** **9**).^[^ ^113^ ^]^ The resulting microcapsules (21.3 ± 2.9 µm) exhibited promising drug‐loading capacity (61.6%), encapsulation efficiency (56%), and promoted reduced inflammatory cell infiltration, increased capillary formation, and organized collagen fiber arrangement in rat dorsal wound models. Initial hemostasis was indeed supported by chitosan, which exhibits inherent blood‐clotting and antibacterial properties, hence reducing the risk of infections that could delay neovascularization.^[^ ^18^ ^]^

Yang et al.^[^ ^114^ ^]^ prepared submicronic capsules (≈0.15–0.36 µm) laden with *Nostoc commune* Vaucher polysaccharide (NCVP), within an alginate/chitosan matrix via emulsion‐crosslinking method.^[^ ^114^ ^]^ These microcapsules exhibited a relatively spherical morphology and porous 3D structure. Excellent biocompatibility and low cytotoxicity in rodent models were proven, accelerating wound closure (≈80% after 14 days) and enhanced angiogenesis. While chitosan is believed to directly interact with red blood cells, facilitating clot formation, alginate absorbs wound exudate creating a gel‐like structure that aids in blood stabilization.^[^ ^60^, ^115^ ^]^ Additionally, in this formulation, the presence of polysaccharides promoted healthy granulation tissue and angiogenesis, in addition to their immunomodulatory potential and antioxidant activities.^[^ ^114^, ^116^ ^]^

Later, the same research group conducted in vivo rat skin trauma experiments.^[^ ^117^ ^]^ It was found that the optimized NCVP capsule formulation could increase the content of hydroxyproline and glutathione to protect cells from oxidative damage, yielding accelerated wound healing (≈93%). Cell division, collagen, and microvascular production were also facilitated by increasing transcription levels of vascular endothelial growth factor messenger RNA (mRNA) and microRNA‐21, likely minimizing the risk of failure to heal.^[^ ^118^ ^]^

Additional reports also advocate that biopolysaccharides (e.g., *Malus halliana* Koehne) exhibit procoagulant activities in vitro, promoting blood clotting via endogenous/exogenous coagulation pathways, improving fibrinogen content, and benefitting blood vessel growth.^[^ ^119^ ^]^

Yang et al.^[^ ^120^ ^]^ engineered spherical microcapsules (≈200 µm), laden with vascular endothelial growth factor (VEGF), featuring an alginate–chitosan matrix.^[^ ^120^ ^]^ These were prepared via polyelectrolyte complexation followed by glutaraldehyde crosslinking, employing a microfluidic electrospray device. Remarkably, VEGF was sustainably released from the microcapsules over several days. After 11 days, the corresponding residual wound area was reduced to ≈5% compared to control (≈40%) and alginate only (≈40%), suggesting that VEGF‐laden microcapsules may accelerate blood clotting. When casted on the wound site, they not only promoted angiogenesis and endothelialization but also dramatically reduced bacterial growth (*E. coli* and *S. aureus*).^[^ ^121^ ^]^

Generally, chitosan may support blood clotting by promoting platelet aggregation and adhesion.^[^ ^122^ ^]^ Its marine variants exhibit higher adaptability and lower immunogenicity when compared to terrestrial animal‐sourced biopolymers, minimizing the risk of disease transmission (zoonosis).^[^ ^123^ ^]^ However, challenges may persist in standardizing production at a large scale. This is mostly due to the source ability of marine chitosan batches (e.g., different geographic origin) and the possible presence of heteroinclusions and/or contaminants, such as micro/nanoscaled plastics.^[^ ^124^ ^]^ For instance, utilizing fungally fermented chitosan may circumvent these limitations, ruling out contamination that could impact human safety and efficacy, and ensuring regulatory compliance.^[^ ^125^ ^]^

Alternatively, Chen et al.^[^ ^126^ ^]^ presented biocompatible microcapsules (1–2 µm) made of glycidyl methacrylate (GMA)‐modified carboxymethyl chitosan hydrogel as a novel tool to tackle hemostasis.^[^ ^126^ ^]^ These microcapsules skillfully incorporated a metal–polyphenol network (MPN), bovine serum albumin, tannic acid, and Fe^3+^, into microbubbles, which enhanced the intramolecular H‐bonding within the hydrogel matrix, yielding a bilayered shell thickness of 30–60 nm, with enhanced mechanical properties. GMA introduced reactive sites for rapid UV‐crosslinking, preserving the antibacterial, anti‐inflammatory, and antioxidant activities.^[^ ^127^ ^]^ The blood coagulation activity of these microcapsules was evaluated in vitro via a blood clot dissolution assay. Interestingly, the new microcapsule‐embedded hydrogel provided superior ability to promote blood clotting (+88%) and effectively prevent blood cells’ dispersion compared to the control group (i.e., pure blood). While the hydrogel shows significant potential as a clinically relevant hemostatic material, challenges may include i) ensuring consistent crosslinking efficiency, ii) optimizing capsule integration for scalable production, and iii) further validating its performance in complex in vivo environments.

Wang et al.^[^ ^128^ ^]^ designed nontoxic carboxymethylcellulose‐based hydrogels featuring curcumin‐laden starch microcapsules, which aided in wound healing by reducing inflammation and preventing infection.^[^ ^128^ ^]^ Interestingly, the combination of CMC hydrogel and microcapsules promoted overall stability and antibacterial effects of the dressing which also exhibited significant mechanical properties (compressive strength ≈ 5.3 MPa; a tensile strength of 2.5 MPa) compared to traditional hydrogel‐based materials (e.g., Young's modulus of chitosan–alginate materials in liquid ≈10–400 kPa).^[^ ^129^ ^]^ However, it seems that the dressing was primarily developed to achieve fast and scarless posthemostatic healing, as neither specific data on its hemostatic efficacy nor blood clotting index were included.

Beşen et al.^[^ ^130^ ^]^ utilized gum Arabic to obtain microcapsules (1–20 µm) with a core of ozonated flaxseed and *St. John's wort* oils via a simple coacervation method.^[^ ^130^ ^]^ These oil‐laden microcapsules, when embedded into medical textiles (e.g., ≈25 × 25 mm four‐ply gauze patch), displayed higher antibacterial (up to 100% bacterial clearance after inoculation at 37 °C over ≈24 h) and enhanced wound‐healing properties. These microcapsules were characterized for thermal stability and effectively contributed to wound contraction. However, despite their remarkable biological adaptation, they did not seem to show any direct clotting activity, likely improving hemostasis indirectly by minimizing the risk of infection and biofilm formation that can delay the later repair stages. Although Beşen et al.^[^ ^130^ ^]^ explored the tensile strength of the microcapsule‐impregnated medical textiles, and others examined the hardness (≈1.3 N), the elastic springiness (which likely refers to the elastic deformation ≈ 0.07 mm), and relative adhesiveness (≈0.03 mJ) of NCVP microcapsules, the majority of publicly available research has overlooked the mechanical properties of individual microcapsules.^[^ ^114^ ^]^ Yet, this is imperative for predicting the performance and efficacy of HWD, when applied to sensitive or sore skin under mechanical stress. Advanced techniques, such as micromanipulation,^[^ ^125^, ^131^ ^]^ offer a promising avenue for characterizing the mechanical properties of microcapsules, for a better understanding of their versatility and potential applications.

Overall, these studies highlight the versatile roles of polysaccharide‐based microcapsules in promoting hemostasis and accelerating wound healing through antibacterial activity, vascular regeneration, and tissue remodeling, rendering them promising candidates for advanced next‐generation hemostatic wound devices. Key microcapsule‐assisted systems are presented in **Table** **2**.

**Table 2:** Microcapsule‐assisted HWDs based on their key functionalities.

Encapsulation method	Functional active ingredient	Wall material	Size [µm]	Properties	Reference
Layer‐by‐layer self‐assembling	*Codonopsis pilosula* polysaccharide	Sodium alginate, calcium chloride, chitosan Glutaraldehyde	21.3 ± 2.9	*C. pilosula* promotes immune regulation; chitosan possesses hemostatic, antimicrobial and antioxidant activities good degradation rate after 11 days (72.8%), loading rate (61.6%), encapsulation rate (56%), maximum swelling rate (397.4 ± 25.3%)	[113]
Complexation followed by crosslinking (microfluidic electrospray)	Vascular endothelial growth factor (VEGF)	Alginate and chitosan	≈200	Shell exhibited antibacterial capacity; VEGF promoted angiogenesis; excellent biocompatibility; dramatic reduction of bacterial growth (*E. coli* and *S. aureus*)	[120]
Emulsion‐chemical crosslinking method	*Nostoc commune* Vaucher polysaccharides	Alginate and chitosan	0.15–0.36 (submicronic)	Microcapsules with spherical morphology; excellent biocompatibility and low cytotoxicity in rodent models; wound closure (≈80% after 14 days); enhanced angiogenesis (key cytokines); chitosan interacts with red blood cells, facilitating clot formation; alginate absorbs wound exudates Accelerated wound healing (≈93%); facilitated cell division and collagen production by increasing transcription levels of vascular endothelial growth factor mRNA and microRNA‐21	[114, 117]
Photo‐crosslinking	Metal (Fe^III^)–polyphenol–tannic acid (TA)‐albumin microbubbles	Glycidyl methacrylate‐modified carboxymethyl chitosan	≈1–2	Bilayered shell thickness of 30–60 nm; promoting blood clotting (+88%); preventing blood cells dispersion; antibacterial, anti‐inflammatory, and antioxidant activity	[126]
Sonochemical methodology	Curcumin	Carboxymethylcellulose (CMC), starch	–	Reducing inflammation and preventing bacterial infection; drug loading (43.2%); encapsulation efficiency (91.3%); CMC‐based hydrogels: remarkable healing ability in in vivo wound‐healing tests	[128]
Coacervation	Ozonated flaxseed and *St. John's wort* oils	Gum Arabic	1–20	High antibacterial activity (up to 100% clearance) against gram positive/negative bacteria; enhanced wound healing (wound area: ≈20% down to 5% within 9–10 days); microcapsules applied to gauzes with padding method	[130]

### 5.6 New Avenues in Capsule‐Assisted Hemostatic Systems

The development of hierarchically structured microcapsules (HSMs) for the augmentation of mesh materials in experimental models of abdominal wall repair demonstrates the potential of microcapsule‐based strategies in multiple biomedical applications.^[^ ^132^ ^]^ HSMs offer a dual‐function approach by incorporating proangiogenic molecules and AgNPs for antibacterial properties.^[^ ^133^ ^]^ HSMs might effectively promote blood vessel formation, reduce bacterial contamination, and enhance tissue regeneration, as shown in defected abdominal tissues.

This concept may be adapted to hemostatic applications, where microcapsules could deliver important hemostatic agents like tranexamic acid in a sustained manner, aiding in faster wound healing and preventing infection.^[^ ^134^ ^]^ Indeed, Tang et al.^[^ ^134^ ^]^ engineered double crosslinked multifunctional gel microarchitectures loaded with tranexamic acid, employing a catechol‐modified hyaluronic acid dopamine/carboxymethyl chitosan matrix. Large water uptake (505.9 ± 62.1%) and rapid in vivo hemostasis (79 ± 4 s) were achieved.^[^ ^134^ ^]^ Advantages of such architectures may include hierarchic, multicore–shell configuration, high monodispersity, tuneable shell thickness for controlled drug release, biocompatibility, and low cytotoxicity.^[^ ^135^ ^]^

An interesting approach was adopted by Nqoro et al.^[^ ^136^ ^]^ who utilized sodium alginate‐based topical gels laden with essential oils, iron oxide nanocarriers, and tranexamic acid to promote hemostasis and observed that 99% of a defect was covered by fibroblasts within 3 days compared to 24% in controls, using an in vitro wound scratch model.^[^ ^136^, ^137^ ^]^ However, challenges remain in optimizing performance properties, a concern that extends beyond clinical and biomedical applications.^[^ ^124^ ^]^ In addition, it is worth noting that the inclusion of essential oils could lead to potential allergenic reactions or irritation in sensitive individuals.^[^ ^138^ ^]^

Exploring multifunctional HSMs for hemostasis may pave a promising avenue for advancing wound care, reducing inflammation, and ensuring the formation of healthy granulation tissue. Future research should focus on tailoring the properties of microcapsules to meet specific hemostatic challenges, improving their stability, and systematically investigating their mechanical and adhesive properties to yield highly performing in situ dressing‐to‐wound hemostatic devices. Additionally, refining controlled release mechanisms will be crucial for enhancing their clinical versatility and overall effectiveness.

Another promising methodology was presented by Thakur et al.^[^ ^139^ ^]^ who developed nanorod systems featuring egg‐shell‐derived calcium and ionic Zn^2+^ from zinc sulfate, which were encapsulated using chitosan polymers to create advanced hemostatic bandages.^[^ ^139^ ^]^ These biocompatible bandages exhibited exceptional blood clotting indices, achieving ≈84–93% clotting within minutes. Moreover, remarkable protein adsorption (≈37 mg cm^−2^) and platelet aggregation (65% within 10 min) were demonstrated, in addition to significant bactericidal activity against *E. coli* and *S. aureus*. Overall, this system relies on positively charged chitosan to enhance clotting, combined with calcium and zinc playing important roles in platelet activation and collagen synthesis, respectively.^[^ ^72^, ^140^ ^]^ However, the economic feasibility of scaling the process and ensuring regulatory compliance for clinical use remain critical considerations.

### 5.7 Composite Sponges

Microporous sponges have emerged as innovative materials for hemostatic applications due to their ability to absorb blood rapidly, promote clotting, and provide a physical barrier at wound sites.^[^ ^141^ ^]^ By incorporating bioactive agents within their porous structure, these sponges can enhance antimicrobial and wound‐healing properties, making them ideal candidates for multifunctional hemostatic wound devices.^[^ ^142^ ^]^ Among these developments, biocompatible gellan gum sponges embedded with ZnO nanoparticles (≈70 nm in diameter) have shown promise for hemostasis and wound healing.^[^ ^143^ ^]^ These sponges exhibited strong antibacterial activity against *E. coli* and *S. aureus*, with a blood absorption capacity (above 75–95%) significantly outperforming commercial gelatine‐based products (10–25%), hence reducing bleeding time. However, ZnO dose‐dependent cytotoxicity may be observed at relatively high ZnO concentrations necessitating the requirement for future investigations to elucidate safety.^[^ ^144^ ^]^

Similarly, Zhou et al.^[^ ^145^ ^]^ produced gelatine–kaolin sponges that significantly reduced hemostasis times in both liver (≈70 s) and femoral artery (≈75 s) models, while minimizing the overall blood loss (219 and 948 mg, respectively).^[^ ^145^ ^]^ The incorporation of kaolin particles likely facilitated rapid clot formation, possibly due to a faster activation of Factor XII.^[^ ^76^ ^]^ These results highlight the potential of combining both natural and animal‐sourced components to enhance hemostatic efficiency.

Goncharuk et al.^[^ ^146^ ^]^ prepared porous poly(vinyl formal)‐based sponges using nanosized aminopropyl silica, microscaled CaCO_3_ (2–5 µm), and chitosan hydrogel as modifying agents.^[^ ^146^ ^]^ These sponges achieved dramatic reduction in blood loss (≈97% compared to traditional gauzes; ≈95% compared to commercial Celox). While inorganic fillers (i.e., silica and CaCO_3_) enhanced the structural–mechanical properties, their contribution to hemostasis was minimal. A key challenge in this study was the decrease in swelling and subsequently the overall hemostatic efficiency when pores became clogged with chitosan. This elucidated the importance of maintaining optimal porosity and active specific surface area for exudate/blood absorption.

Chinese herbal compounds, such as *B. Striata*, have also garnered attention for their promising biocompatibility, high fluid absorbability, and hemostatic properties. These were successfully used in combination with zeolites (see Section 4.1),^[^ ^78^ ^]^ as well as crosslinked with chitosan,^[^ ^147^ ^]^ and porous alginate‐calcium to produce styptic sponges.^[^ ^148^ ^]^ For instance, Han et al.^[^ ^147^ ^]^ demonstrated that *B. striata* sponges were capable of achieving blood clotting in ≈150 s (blood loss ≈ 13 mg), significantly outperforming untreated group (blood loss ≈ 31 mg; time to hemostasis ≈ 430 s) in healthy tail‐amputation mouse model.^[^ ^147^ ^]^ Moreover, strong antibacterial and anti‐inflammatory properties were reported, while promoting cell proliferation and collagen deposition. Interestingly, Enzyme Linked Immunosorbent Assay (ELISA)‐assisted mouse wound experiments in the presence of these sponges, improved vascular endothelial growth factor and demonstrated that inflammatory markers, such as tumor necrosis factor‐alpha (TNF‐α) and interleukin‐6 (IL‐6), were reduced.

On a similar note, Wang et al.^[^ ^148^ ^]^ fabricated sponge systems using *B. striata*–chitosan while incorporating alginate–calcium porous microparticles (158 µm, pores size 200–300 nm).^[^ ^148^ ^]^ These sponges effectively improved water absorption (≈0.05 g) and facilitated rapid clotting by concentrating coagulation factors. In vitro and in vivo tests revealed that the time to hemostasis was as low as ≈56 s with minimal blood loss (0.023 g), outperforming commercial gelatine sponges (blood loss ≈ 0.05 g). Although sponges containing *B. striata* exhibited anti‐inflammatory activity and excellent biocompatibility, challenges persist, such as ensuring consistent quality during production and achieving cost‐effective scalability for mass manufacturing.^[^ ^149^ ^]^ Furthermore, optimizing the mechanical strength of the composite sponge is paramount to expand its applicability across a broad spectrum of surgical procedures.

Innovative composite sponges were formulated using modified starch and halloysite nanotubes (mean particle size of around 320 nm) for addressing noncompressible hemorrhage.^[^ ^150^ ^]^ The nominal compressive stress of the sponge was twofold higher compared to the corresponding controls, while reducing clotting time by over 50%. In severe bleeding scenarios, such as rabbit carotid artery transection, blood loss was reduced by about 20 g within 5 min. Despite these promising results, challenges include potential variability in performance due to possible differences in wound types or bacteria‐rich environments; moreover, further testing is needed to confirm long‐term safety and efficacy in emergency settings.

In a similar vein, Zhou et al.^[^ ^151^ ^]^ explored hydrophilic Mxene (a class of 2D materials composed of transition metal carbides, nitrides, or carbonitrides) to achieve multifunctional sponges, for wound management.^[^ ^151^ ^]^ These sponges demonstrated superior antibacterial activity (>97%) against *E. coli* and *S. aureus*, large water absorption (39 times), and high porosity (91%). Remarkably, endogenous electric fields were leveraged to direct cell migration and promote tissue repair, improving collagen deposition, angiogenesis, and re‐epithelialisation while minimizing scarring. In hemostasis assays via rat tail‐amputation model, these sponges achieved accelerated blood clotting (≈95 s) compared to conventional collagen sponges (≈120 s). However, concerns over the long‐term biocompatibility and scalability of Mxene sponges persist, particularly given the use of glutaraldehyde as a crosslinking agent.

In addition, shape memory hydrogel sponges hold potential for controlling bleeding in noncompressible and deep wounds.^[^ ^152^ ^]^ Succinic ester‐based sponges were developed by Zhu et al.^[^ ^152^ ^]^ via cryopolymerisation, yielding relatively rapid hemostasis (0.4 min) and significantly reduced blood loss (≈66  mg) in animal models, compared to commercial gelatine sponges (1.8 min, ≈133 mg), owing to superior fluid absorption and red blood cell affinity.

Despite their promise, key challenges remain. Long‐term biocompatibility, controlled degradation, and clear understanding of procoagulant mechanisms require further study. Moreover, issues such as storage stability, usability, and clinical scalability must be addressed to realize their translational potential.

Overall, microporous sponges represent a versatile tool for advanced hemostatic and wound‐healing management. Future research should focus on optimizing biocompatibility, mechanical strength, and large‐scale manufacturing, while addressing the challenges associated with specific bioactives. The development of multifunctional sponges capable of addressing complex surgical/trauma scenarios remains an exciting frontier in medical material innovation.

### 5.8 Regulatory Compliance for Potential Clinical Translation

The clinical translation of particle‐based hemostatic wound devices is challenged by complex frameworks stemming from their hybrid nature, which often combines pharmaceutical agents, structural matrices, and biological or synthetic delivery systems. This typically requires compliance with multiple regulatory bodies, such as the U.S. Food and Drug Administration (FDA) and the European Medicines Agency (EMA), whose differing standards can hinder product development and international harmonization. While significant strides have been made in the development of advanced preclinical model systems, current translational methodologies remain insufficient to support the streamlined development of personalized medical devices, including micro/nanoparticle‐assisted HWDs.^[^ ^153^ ^]^

A major bottleneck lies in the lack of standardized pharmacokinetic, pharmacodynamic, and preclinical testing protocols tailored to particulate systems in wound environments. Existing models often lack clinical relevance, limiting predictive accuracy.^[^ ^154^ ^]^ Consequently, manufacturers are compelled to undertake customized, resource‐intensive studies to demonstrate safety, efficacy, and long‐term biocompatibility, substantially escalating both timelines and costs. Additional challenges arise from the inherent sensitivity of particle‐integrated systems to batch‐to‐batch variability, environmental factors, and processing‐induced stress, such as shear‐driven aggregation.

Sterilization also represents another critical, yet overlooked, barrier to clinical translation. Conventional methods, such as gamma irradiation, ethylene oxide, and steam autoclaving, can significantly alter the physicochemical properties of particles and hydrogels, including surface charge, morphology, drug release kinetics, and biological activity, compromising hemostatic function and/or safety.^[^ ^155^ ^]^ Future research should focus on the development of sterilization approaches that effectively preserve both structural and functional integrity of particles and hydrogels, while being scalable and compliant with regulatory standards.

## 6 Multifunctionalized Hydrogels

Multifunctionalized hydrogels have emerged as versatile delivery platforms in advanced wound care, particularly due to their tuneable physical properties, biocompatibility, and capacity to mimic the extracellular matrix.^[^ ^156^ ^]^ They can serve as structural bioscaffolds for microparticles and nanoengineered systems, enabling the sustained release of therapeutic agents, targeted hemostatic action, facilitating tissue regeneration.^[^ ^157^ ^]^ Embedding particle‐based technologies within hydrogel matrices creates synergistic systems that respond dynamically to wound environments. In addition, hydrogels address the limitations of conventional hemostatic agents, such as biotoxicity and poor degradability, by utilizing biocompatible components, such as animal‐sourced materials (e.g., chitosan, gelatine, and homotrimer/heterotrimer collagens)^[^ ^158^ ^]^ and other plant‐based, biomimetic derivatives.^[^ ^6^ ^]^

Song et al.^[^ ^159^ ^]^ developed an adhesive hydrogel consisting of quaternized chitosan–methacryloyl, polyvinylpyrrolidone, and dopamine.^[^ ^159^ ^]^ This hydrogel demonstrated impressive swelling ratio (up to ≈3000% in water, and ≈2000% in phosphate buffer saline (PBS) and 0.9% NaCl physiological solution) and reversible adhesion properties (12–25 kPa), ensuring effective sealing of bleeding tissues even in wet environments. In Sprague–Dawley rat models, it significantly reduced blood loss by ≈76% following tail amputation, showcasing its clinical potential. Although this hydrogel represents a promising yet evolving solution against wound bleeding, its effectiveness in human trials and long‐term biocompatibility may require further exploration to fulfill clinical needs.

**Figure 10:**
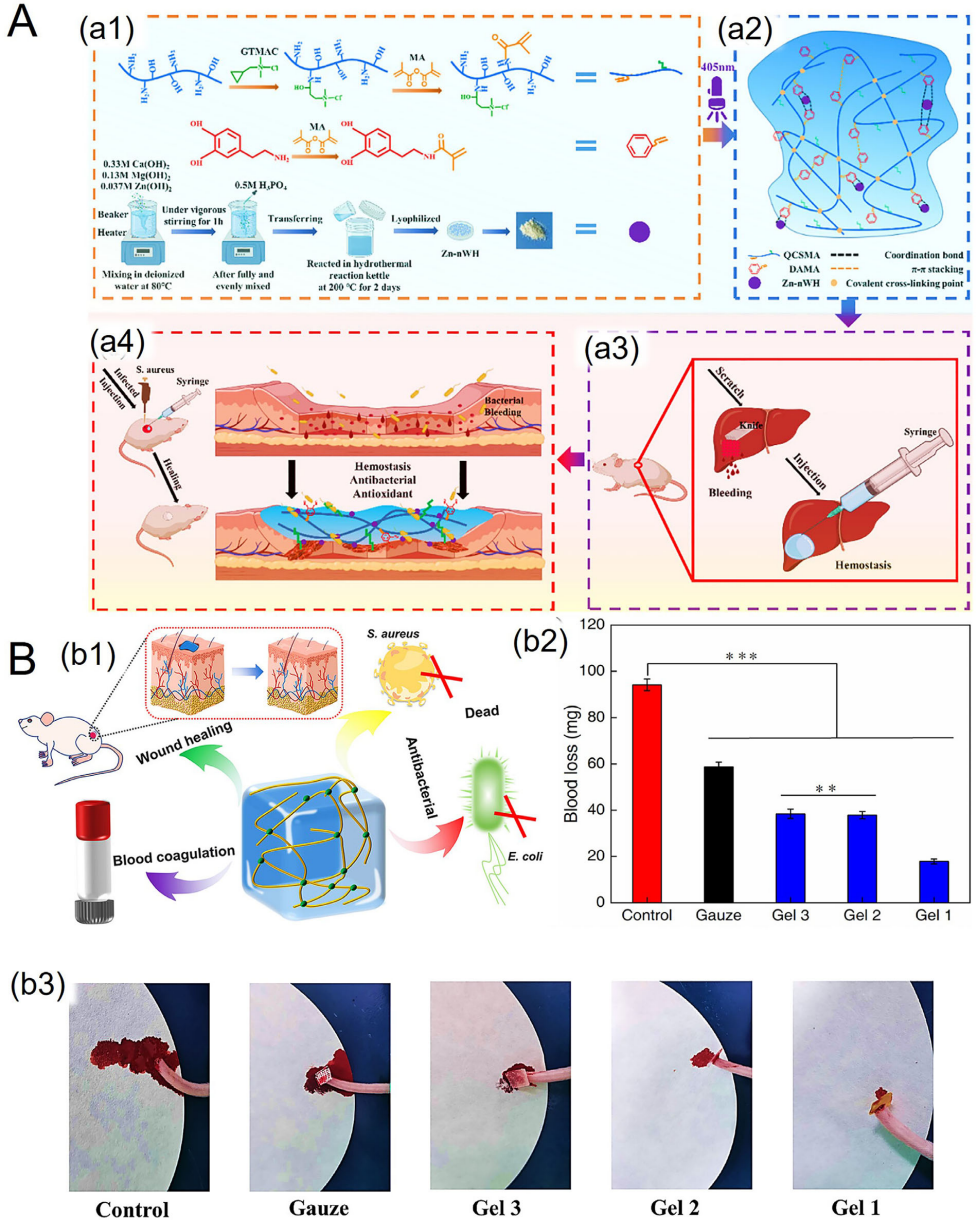
A) Synthesis process of a1) quaternized chitosan methacrylate anhydride (QCSMA), dopamine methacrylate anhydride (DAMA), and zinc‐doped whitlockite (Zn‐nWH) nanoparticles; a2) schematic representation of the hydrogel formation by incorporating QCSMA, DAMA, and Zn‐nWH nanoparticles; a3) application of the hydrogel for liver hemostasis; and a4) application of the hydrogel for infected wound healing. Reproduced with permission.^[^ ^160^ ^]^ Copyright 2022, Elsevier. B) Schematic representation of the reaction mechanism involved in b1) hydrogel preparation and its end‐use applications; b2) quantitative results from hemostasis experiments in a mouse tail‐amputation model in the presence of three hydrogel variants (error bars represent the standard deviation); and b3) images of hemostasis experiments conducted in the mouse tail amputation model. Reproduced with permission.^[^ ^164^ ^]^ Copyright 2024, American Chemical Society.

Multifunctional hydrogels engineered with methacrylate anhydride dopamine, zinc‐doped whitlockite nanoparticles, and methacrylate anhydride quaternized chitosan, showed promise in hemostasis and wound healing (**Figure** **10**A).^[^ ^160^ ^]^ This is due to their rapid gelation, strong tissue adhesion (>0.03 MPa), advantageous biocompatibility, no cytotoxicity, and a hemolysis ratio under 2%. Indeed, these hydrogels demonstrate exceptional hemostatic performance, reducing bleeding times by ≈80% (129 ± 22 s compared to 571 ± 15 s in controls), alongside over 90% bactericidal effectiveness against *S. aureus* and *E. coli*. However, challenges like production scalability and cost efficiency should be further investigated for widespread clinical use.^[^ ^161^ ^]^

In the same light, Song et al.^[^ ^162^ ^]^ fabricated multifunctional chitosan‐based biohydrogels with remarkable properties, including stretchability (up to 780%), exceptional blood absorption (1300 ± 50%), and strong adhesion (68.5 kPa) to various biomimetic substrates (e.g., pig skin).^[^ ^162^ ^]^ This ensures broad‐spectrum versatility and in situ hemostatic activity on wounds under dynamic conditions, due to its swift clotting time (50 s) and lower blood clotting index (41), which outperforms many classic hemostatic materials. While the self‐healing (Schiff base), antibacterial, and biocompatible properties enhance their potential for emergency applications, long‐term biodegradation effects should be further investigated. Detailed insights into green and sustainable hydrogels based on quaternized chitosan for enhanced wound healing have been provided by Mottaghitalab et al.^[^ ^163^ ^]^

Dong et al.^[^ ^164^ ^]^ designed a multi‐inclusional antibiotic‐free hydrogel, composed of dialdehyde cellulose, l‐glutamine, iodine, and 2‐hydroxypropyl‐β‐cyclodextrin (HP‐β‐CD) (Figure 10B).^[^ ^164^ ^]^ This hydrogel leveraged iodine's potent efficacy against pathogens like *E. coli* and *S. aureus*, while the Schiff base triggered by l‐glutamine synergistically promotes tissue regeneration and bactericidal effects.^[^ ^165^ ^]^ The modification of cellulose by polar l‐glutamine/HP‐β‐CD‐enhanced blood coagulability in vitro and dramatically shortened its clotting window. Notwithstanding, challenges may remain in ensuring scalability and cost‐effectiveness for clinical applications.

Similarly, Li et al.^[^ ^166^ ^]^ developed injectable, biocompatible hydrogels based on carboxymethyl cellulose‐graft‐adipic dihydrazide and 4‐formylbenzoic acid‐terminated poly(ethylene glycol) for rapid hemostasis and wound healing.^[^ ^166^ ^]^ In addition to their strong adhesive properties to irregular wound surfaces, likely due to the presence of hydrogen bonding and Schiff bases,^[^ ^167^ ^]^ these hydrogels provided a moist, antibacterial environment, releasing ciprofloxacin to combat infections. Importantly, they promoted quick clot formation and subsequent angiogenesis: in in vivo hemorrhaging liver mouse model, blood loss was reduced by around 80% (control ≈450 mg; hydrogel ≈80 mg), evidencing strong hemostatic properties. Yet, potential challenges include ensuring consistent in vivo biodegradability, and addressing cytotoxicity risks from aldehydic residues.

Another approach was proposed by Fang et al.^[^ ^168^ ^]^ who produced bio‐inspired hydrogels, composed of catechol‐functionalized quaternized chitosan and dibenzaldehyde‐terminated polyethylene glycol.^[^ ^168^ ^]^ Their hydrogels demonstrated rapid gelation (17.60 ± 0.65 s), strong tissue adhesion, and effective antibacterial and antioxidant properties. Interestingly, rapid in vivo hemostasis in rat liver and carotid wounds was proven, while promoting collagen deposition and angiogenesis in methicillin‐resistant *S. aureus*‐infected skin injuries. Further studies should be conducted to evaluate long‐term stability, and the potential for clinical translation across broader wound types.

Interestingly, chitosan‐based copper nanoparticles integrated with zinc oxide hydrogel demonstrated significant antibacterial activity against both Gram‐positive and Gram‐negative bacteria.^[^ ^169^ ^]^ The nanoparticles (1–100 nm) enhanced in vivo wound healing within 16 days by promoting cell proliferation and granulation tissue formation. Histological analysis showed that the rat cohort treated with this formulation underwent higher leukocyte infiltration and larger degrees of collagen deposition.

Recently, Li et al.^[^ ^1^ ^]^ formulated a multifunctionalized hydrogel, incorporating γ‐polyglutamic acid, polyethyleneimine, and montmorillonite.^[^ ^1^ ^]^ Specifically, γ‐polyglutamic facilitates rapid absorption of aqueous fluids (e.g., exudate),^[^ ^170^ ^]^ while the uniform distribution of montmorillonite─NH_2_ imparts dual benefits: i) strengthening the maximum mechanical compressive stress of the hydrogel (1692.8 ± 141.4 kPa compared to montmorillonite‐free hydrogel 244.8 ± 7.3 kPa),^[^ ^171^ ^]^ and promoting rapid blood coagulation. Based on in vivo hemorrhage simulated experiments (rat tail vein), the multifunctional hydrogel demonstrated markedly reduced blood loss (0.06 ± 0.01 g), compared to the nonfunctionalized hydrogel (0.70 ± 0.03 g). Additionally, the modification of polyethyleneimine with 3,4‐dihydroxybenzaldehyde enhanced the anti‐inflammatory and antioxidant properties of the hydrogel, fostering an optimal environment for wound healing.^[^ ^172^ ^]^ However, despite these promising features, this formulation exhibited limited adhesion to wet tissues due to the absence of specific functional groups, such as aldehyde (─CHO) groups, possibly restricting its effectiveness in broader clinical scenarios.

## 7 Stimuli‐Responsive HWDs

Stimuli‐responsive wound dressings have emerged as an innovative drug‐eluting approach, designed to respond to specific internal or external triggers, such as pH changes, temperature variations, glucose, light, or magnetic fields.^[^ ^6^, ^173^ ^]^

Other than chronic wounds, such as diabetic ulcers,^[^ ^174^ ^]^ novel stimuli‐responsive formulations have been reported in the literature to facilitate wound hemostasis. For example, Shi et al.^[^ ^105^ ^]^ developed a magnetic‐guided hemostatic system using microporous starch particles, iron oxide, and thrombin, achieving significant advantages in targeting complex bleeding wounds.^[^ ^105^ ^]^ The system halts bleeding rapidly, with hemostatic times of 37 s for liver V‐shaped wounds and 44 s for J‐shaped wounds, surpassing traditional hemostats like Celox, which required 121 s. Furthermore, its biodegradability in subcutaneous muscles was achieved within 84 days, with minimal cytotoxicity. However, challenges may include ensuring consistent performance in varied clinical settings, potential regulatory hurdles, and the need for specialized equipment to generate precise magnetic fields.

Similarly, Liu et al.^[^ ^175^ ^]^ demonstrated a promising photoresponsive material for smart hemostasis, leveraging an azobenzene‐containing surfactant to control the release of protamine for neutralizing heparin‐driven anticoagulation.^[^ ^175^, ^176^ ^]^ This enabled cationic and anionic proteins to function as phase‐change biomaterials, transitioning into an isotropic state through UV–Vis irradiation.^[^ ^175^ ^]^ The photo‐responsive system showed superior hemostatic performance, with UV‐treated complexes reducing blood loss in a murine tail‐cut model by 37.9% and hemostatic time by 29.4% compared to pure protamine. This innovation enables spaciotemporal control of heparin scavenging, with the blood clotting index decreasing to ≈21% upon UV treatment. Notwithstanding, concerns about potential tissue damage from UV exposure and the complexity of practical application may remain.

Preman et al.^[^ ^177^ ^]^ highlighted a novel dual‐stimulus (pH–temperature)‐responsive hydrogel formulated from sodium alginate and poly(*N*‐vinyl caprolactam), with Ca^2+^ and tannic acid as ionic co‐crosslinking agents.^[^ ^177^ ^]^ The resulting hydrogel‐based scaffold excelled in hemostasis, antibacterial action, and promoting cell proliferation, in addition to its promising temperature‐dependent mechanical properties (moduli *G*′ and *G*″ significantly increased from 43 °C). Specifically, its hemostatic effectiveness was significant, reducing blood loss in a murine liver injury model from 0.5 g in the control groups to just 0.1 g within 3 min. This was likely owed to tannic acid reacting with blood proteins, and trigger rapid coagulation.^[^ ^178^ ^]^

Self‐adaptive wound dressings with multiple stimuli‐responsiveness and bactericidal activity were developed by Yang et al.^[^ ^179^ ^]^ employing nanozyme‐based cryogels. These cryogels exhibited rapid hemostasis, with a bleeding time of ≈198 s, significantly outperforming previous gelatine sponges (≈334 s) and the blank groups (≈482 s) in murine liver injury models.^[^ ^179^ ^]^ Their pH‐responsiveness aided bacterial capture and infection management, while near‐infrared (NIR) irradiation triggered the release of nitric oxide (NO),^[^ ^180^ ^]^ promoting photodynamic and photothermal activity against bacteria (*E. coli*).

Overall, it is understood that these systems are designed to promote clot formation, skillfully integrating stimuli‐responsive materials and molecules to stabilize the wound, preventing wound relapse and further blood loss. Yet, potential scalability, cost, and ensuring reproducibility across varied clinical conditions remain to be investigated.

## 8 Conclusions and Outlook

Hemostatic dressings have seen remarkable progress over the past few decades, yet achieving rapid, reliable, and safe hemorrhage control remains a critical challenge in both trauma and surgical care. Commercial hemostatic devices, such as QuickClot Combat and Celox, are clinically employed. However, these devices face significant limitations in managing high‐pressure bleeding, as they often fail to promote and stabilize clots effectively. Furthermore, they require frequent mechanical changes, triggering risks of ‘vicious cycle’ rebleeding. Recent research has focused on addressing these limitations through innovations in particle‐assisted systems, including the integration of biocompatible nanoparticles, and nanobioengineered molecules designed to accelerate coagulation. Important advances include the development of materials that conform to irregular wound geometries, stimuli‐responsive systems that activate upon contact with blood, and formulations that combine both procoagulant and bactericidal agents.

Nonetheless, physical properties of hemostatic dressings remain a critical consideration. The ability to apply effective pressure and conform to wound surfaces is paramount for controlling hemorrhage, especially in cases of severe, “torrential” bleeding. Future research in hemostatic technologies should hinge on the design of versatile, multifunctional, and bioresponsive materials, capable of addressing the complex physiological conditions associated with acute bleeding in prehospital settings. Prioritized studies should centre on the development of flexible, ultralight, highly absorbent, conformable matrices, featuring precision‐targeted delivery systems for localized modulation of the coagulation cascade, enhancing both the speed and efficacy of hemostasis.

Novel approaches, such as micro/nanocapsule‐integrated devices that harness stimuli‐responsive properties and/or enable controlled release of procoagulant agents, could represent a transformative leap in this field. Furthermore, integrating biosensing platforms into hemostatic devices, possibly coupled with artificial intelligence (AI)‐driven tools, could enable monitoring of bleeding dynamics, clot formation, and biomarker feedback. This would support intelligent, personalized, therapeutic trauma management, assessing bleeding severity and clotting progress and in real time.

From a translational perspective, economic factors and market dynamics should not be overlooked, as the global demand for hemostatic products continues to rise. Emphasis should be placed on scalable, low‐cost fabrication methods that retain the functional complexity of advanced systems, which is essential to meet growing clinical needs, particularly in low‐resource healthcare settings.

In conclusion, while technical and regulatory challenges persist, the trajectory of innovation in particle‐assisted styptic devices is highly promising. The development of versatile biomaterials and advanced hemostatic technologies has the potential to revolutionize trauma care. Future research can help usher in a new paradigm in hemorrhage control, and ultimately save countless lives, by focusing on programmable materials, delivery platforms, integrated biosensing, and translational scalability, defined by precision, adaptability, and global accessibility.

## Conflict of Interest

The authors declare no conflict of interest.
